# Chemical multiplexing in the nervous system: molecular architecture, functional stratification, and pathophysiological plasticity of neuropeptide-classical neurotransmitter cotransmission

**DOI:** 10.3389/fnmol.2026.1839947

**Published:** 2026-06-22

**Authors:** Adalberto Merighi, Marco Sbriz, Laura Lossi

**Affiliations:** Department of Veterinary Sciences, University of Turin, Turin, Italy

**Keywords:** circuit dynamics, cotransmission, dense-core vesicles, neural coding, neuromodulation, neuropeptides, synaptic plasticity, volume transmission

## Abstract

Neurons frequently synthesize both neuropeptides and classical low–molecular-weight neurotransmitters, challenging the historical interpretation of one-neuron-one-transmitter signaling. Rather than representing biochemical redundancy, coexistence reflects a conserved organizational strategy that expands neural coding capacity through chemical multiplexing. Dual vesicular architecture–small clear vesicles for rapid synaptic transmission and large dense-core vesicles for activity-dependent peptide release–creates stimulus-intensity-dependent recruitment of distinct signaling layers. Classical transmitters primarily mediate millisecond-scale synaptic precision, whereas peptides engage slower G-protein–coupled receptor pathways that modulate excitability, plasticity, gene expression, and neuron–glia interactions. Across mammalian circuits, cotransmission regulates oscillatory coherence, learning and memory, motivational states, endocrine integration, sensory gain control, and autonomic balance. Peptidergic signaling is transcriptionally regulated and dynamically remodeled during development, stress, injury, and disease. Dysregulation contributes to chronic pain, addiction, stress-related disorders, epilepsy, cardiovascular dysfunction, and neurodegeneration. Therefore, neuropeptide-neurotransmitter coexistence constitutes a core computational principle of the nervous system, enabling temporal stratification and adaptive plasticity without expanding anatomical connectivity. Understanding chemical multiplexing is essential for linking molecular dynamics to circuit stability and vulnerability in health and disease.

## 1 Introduction - from Dale’s principle to molecular multiplexing: reframing chemical neurotransmission

The classical formulation of chemical neurotransmission emerged from early 20th-century physiology. These physiological studies led to the identification of acetylcholine (ACh) by Loewi (1921), noradrenaline (NA) by von Euler (1946), and adrenaline, originally identified as an adrenal hormone by Abel (1899) and Takamine (1901), as the first classical neurotransmitters in the nervous system. The concept of chemical neurotransmission crystallized into the widely cited “one neuron-one transmitter” interpretation of Dale’s 1934 lecture (Dale, 1935), later reinforced by electrophysiological analyses from John Carew Eccles and colleagues (Eccles, 1957, 1964). Although historically influential, this conceptual simplification obscured a now-fully recognized central property of neuronal communication that is equally fundamental: most neurons produce and release a cocktail of messengers.

Neuropeptides were identified before their formal designation, even before the idea of chemical signaling was established in physiology (Burbach, 2011). During the 19th century, as experimental life sciences advanced, organ extracts were systematically examined for their effects on physiological systems, and the foundations of endocrinology were established. Compounds such as secretin, insulin, vasopressin (AVP), and oxytocin (OT) were identified as having biological activity in crude extracts, as evidenced by bioassays conducted in whole organisms or organ systems (Oliver and Schäfer, 1895; Bayliss and Starling, 1902; Farini, 1913; von den Velden, 1913). In 1905, the English physiologist Ernest Starling coined the term “hormones” to designate such chemicals. It was only in the 1950s and thereafter that the chemical identities of hormones were clarified; notably, nearly all were found to be short chains of amino acids, specifically peptides. Extensive purification of releasing factors from hypothalamic extracts was conducted, leading to an understanding of their structures in the late 1960s and culminating in the awarding of the Nobel Prize to Roger Guillemin, Andrew Viktor Schally, and Rosalyn Yalow in 1977^1^. Beginning in the late 1950s, Victor Mutt identified numerous novel peptide hormones originating from gastrointestinal organs and, subsequently, from the brain, based on their chemical characteristics (Tatemoto and Mutt, 1980). In parallel with the recognition of peptide hormones, another milestone leading to today’s definition of neuropeptides pertains to neurosecretion. Following early research by Carl Speidel in 1917, Ernst Scharrer introduced the notion of neurosecretion (Scharrer, 1954). The peptides identified were AVP and OT, along with their neurophysins, which were subsequently confirmed by immunocytochemistry. The neurosecretion of peptidergic compounds is nowadays recognized as a crucial element of neuropeptide physiology (Hökfelt et al., 1980a). A last aspect in the history of neuropeptides is the identification of their biological effects on the nervous system. David de Wied was a pioneer in studying the effects of peptide hormones on rat behavior from the 1950s, discovering that corticotropin (ACTH), melanocyte-stimulating hormone (MSH), and AVP influenced brain function and impacted learning and memory processes (De Wied, 1971; De Wied and Versteeg, 1979). In the 1970s, he introduced the term “neuropeptides” to refer to neuroactive peptide hormones and their fragments. Today, NeuroPep 2.0, a database dedicated to annotating neuropeptides and receptors, contains 11,417 unique neuropeptide entries across 924 species (Wang et al., 2024). For those unfamiliar with the topic, Table 1 provides an overview of the major peptidergic neurotransmitter systems in the mammalian nervous system. Further information can be found in a forthcoming encyclopedia entry (Merighi, 2026).

**TABLE 1:** Overview of major peptidergic neurotransmitter systems.

Peptidergic system	Main peptides	Gene/precursor	Receptor classes	Coexistence examples	Co-transmission examples
Opioid	β-Endorphin, enkephalins (Met-, Leu-), DYNs, nociceptin/orphanin FQ (Akil et al., 1984; Kieffer and Gavériaux-Ruff, 2002)	POMC (β-endorphin); PENK (enkephalins); PDYN (DYNs); PNOC (nociceptin) (Akil et al., 1984)	μ (MOR), δ (DOR), κ (KOR), NOP/ORL1 (Kieffer and Gavériaux-Ruff, 2002)	Enkephalin co-localizes with GABA in striatopallidal MSNs (Le Moine and Bloch, 1995); DYN with SP in striatonigral neurons (Anderson and Reiner, 1990)	Arcuate POMC neurons co-release β-endorphin + ACTH (Harno et al., 2018); spinal enkephalinergic interneurons co-release enkephalin + GABA to gate nociception (Todd, 2010)
Tachykinin	SP, NKA, NKB, neuropeptide K, neuropeptide γ (Severini et al., 2002)	TAC1 (SP, NKA); TAC3 (NKB) (Severini et al., 2002)	NK1R, NK2R, NK3R (Severini et al., 2002)	SP co-localizes with 5-HT in raphe neurons (Sergeyev et al., 1999); with GABA in cortical interneurons (Jakab et al., 1997); NKB with kisspeptin and DYN (KNDy neurons) (Goodman et al., 2013)	Dorsal root ganglion C-fibers co-release SP + CGRP + glutamate (Russell et al., 2014); KNDy neurons co-release kisspeptin, NKB, DYN to pulse-generate GnRH (Uenoyama et al., 2021)
CGRP	α-CGRP, β-CGRP, AM, amylin (Russell et al., 2014)	CALCA (α-CGRP); CALCB (β-CGRP); ADM; IAPP (amylin) (Russell et al., 2014)	CLR/RAMP1 (CGRP-R), CLR/RAMP2 (AM1-R), CLR/RAMP3 (AM2-R), CTR/RAMP (AMY-R) (Russell et al., 2014)	α-CGRP co-localizes with SP in nociceptive primary afferents (Uenoyama et al., 2021), with ACh at the NMJ (Matteoli et al., 1988)	Trigeminal sensory neurons co-release CGRP + SP + glutamate during migraine-associated neurogenic inflammation (Wrobel Goldberg and Silberstein, 2015); NMJ motor terminals co-release ACh + CGRP to modulate receptor clustering (Di Angelantonio et al., 2003)
NPY family	NPY, PYY, PP (Blomqvist and Herzog, 1997)	NPY; PYY; PPY (Blomqvist and Herzog, 1997)	Y_1_R–Y_5_R (Blomqvist and Herzog, 1997)	NPY co-localizes with NA in locus coeruleus and sympathetic neurons (Illes and Regenold, 1990), with GABA in cortical and hippocampal interneurons (Comeras et al., 2021)	Sympathetic vasoconstrictor terminals co-release NA + NPY (Pernow, 1988); post-synaptic Y_1_R activation potentiates vasoconstriction (Christiansen et al., 2018); hippocampal NPY/GABA interneurons suppress excitability during high-frequency firing (Magloire et al., 2019)
Hypothalamic releasing/ inhibiting peptides	CRH, TRH, GHRH, SST, GnRH, OT, AVP (Russell, 2018)	CRH; TRH; GHRH; SST; GNRH1; OXT; AVP (Russell, 2018)	CRHR1/2, TRHR, GHRHR, SSTR1–5, GnRHR, OXTR, V1aR/V1bR/V2R (Russell, 2018)	CRH co-localizes with AVP in parvocellular PVN neurons (Herman and Tasker, 2016); SST with NPY in cortical interneurons (Herman and Tasker, 2016); OT with DYN in magnocellular neurons (Villar et al., 1990)	PVN parvocellular neurons co-release CRH + AVP synergistically onto pituitary corticotrophs (Itoi et al., 1999); magnocellular OT neurons co-release OT + DYN for autocrine feedback (Hirasawa et al., 2004)
VIP/PACAP family	VIP, PACAP-27, PACAP-38, PHI/PHM, Secretin, Glucagon, GLP-1/2, GIP (Vaudry et al., 2009)	VIP; ADCYAP1 (PACAP); SCT; GCG (Vaudry et al., 2009)	VPAC1R, VPAC2R, PAC1R (Vaudry et al., 2009)	VIP co-localizes with ACh in autonomic parasympathetic neurons (Eva et al., 1985); PACAP with catecholamines in adrenal chromaffin cells (Kuri et al., 2009); VIP with GABA in cortical interneurons (VIP + disinhibitory circuits) (Apicella and Marchionni, 2022)	Parasympathetic neurons to salivary glands co-release ACh (vasoconstriction at low frequency) + VIP (vasodilation at high frequency) – classic frequency-dependent co-transmission (Eva et al., 1985); cortical VIP interneurons co-release VIP + GABA to disinhibit pyramidal cells (Apicella and Marchionni, 2022; Pi et al., 2013)
CCK	CCK-8, CCK-33, CCK-58 (Rehfeld, 2017)	CCK (Rehfeld, 2017)	CCK1R (CCK-AR), CCK2R (CCK-BR/gastrin-R) (Rehfeld, 2017)	CCK co-localizes with DA in mesolimbic neurons (Crawley et al., 1984); with GABA in hippocampal basket cells and cortical interneurons (Pelkey et al., 2017)	Ventral tegmental area neurons co-release DA + CCK (Martinez Damonte et al., 2023); CCK potentiates DA signaling via CCK2R (Martinez Damonte et al., 2023); hippocampal CCK + basket cells co-release CCK + GABA for perisomatic inhibition modulation (Freund and Katona, 2007)
Galanin	Galanin, galanin-like peptide (GALP), alarin (Lang et al., 2015)	GAL; GALP (Lang et al., 2015)	GALR1, GALR2, GALR3 (Lang et al., 2015)	Galanin co-localizes with ACh in BFCNs (McDonald and Crawley, 1997); with 5-HT in dorsal raphe (Ögren et al., 2007); with NA in locus coeruleus (Caestecker et al., 2025)	BFCNs co-release ACh + galanin (Mufson et al., 2005); GALR activation inhibits ACh release (autoinhibitory loop) (Counts et al., 2006); upregulated in AD, possibly compensatory (Counts et al., 2006)
Orexin/ hypocretin	Orexin-A (hypocretin-1), orexin-B (hypocretin-2) (de Lecea et al., 1998)	HCRT (de Lecea et al., 1998)	OX1R (HCRTR1), OX2R (HCRTR2) (de Lecea et al., 1998)	Orexin co-localizes with DYN and glutamate in lateral hypothalamic neurons (Muschamp et al., 2014), with NPTX2 (Reti et al., 2002)	Lateral hypothalamic orexin neurons co-release orexin + DYN + glutamate (Krauth, 2025); glutamate provides fast excitation while orexin sustains arousal (Sakurai, 2007); DYN provides postsynaptic feedback inhibition (Sakurai, 2007)
SST	SST-14, SST-28, cortistatin (Patel, 1999)	SST; CORT (cortistatin) (Patel, 1999)	SSTR1–SSTR5 (Patel, 1999)	SST co-localizes with GABA in cortical and hippocampal interneurons (Martinotti cells) (Kecskés et al., 2020); with NPY in hypothalamic neurons (Anderson and Reiner, 1990)	Cortical Martinotti cells co-release SST + GABA (Wang et al., 2004); SST inhibits glutamate release presynaptically via SSTR, while GABA mediates postsynaptic inhibition – dual suppression of pyramidal output (Kawaguchi and Kubota, 1997)
Neurotensin	NT, neuromedin N (Carraway and Leeman, 1973)	NTS (Carraway and Leeman, 1973)	NTS1R, NTS2R, NTS3R/sortilin (Vincent, 1995)	NT co-localizes with DA in mesolimbic/nigrostriatal pathways (Perez-Bonilla et al., 2020); with GABA in striatum (Patel et al., 2024)	Mesolimbic DA neurons co-release DA + NT (Binder et al., 2001); NT potentiates DA-evoked locomotion via NTS1R (Binder et al., 2001); implicated in antipsychotic-like effects (Goldstein and Deutch, 1992)
Natriuretic peptides	ANP (ANF), BNP, CNP, DNP (Potter et al., 2009)	NPPA (ANP); NPPB (BNP); NPPC (CNP) (Potter et al., 2009)	NPR-A (GC-A), NPR-B (GC-B), NPR-C (Potter et al., 2009)	NPR-C co-localizes with glutamate in hippocampal interneurons (Decker et al., 2009)	Hippocampal interneurons co-release CNP + glutamate (Decker et al., 2009); CNP acts via NPR-B to modulate synaptic plasticity (Decker et al., 2010); cardiovascular neurons co-release ANP + catecholamines (Tota et al., 2010)
Melanocortins	α-MSH, β-MSH, γ-MSH, ACTH, β-endorphin (Mercer et al., 2013)	POMC (Mercer et al., 2013)	MC1R–MC5R (Mercer et al., 2013)	POMC neurons in the arcuate nucleus co-express α-MSH, ACTH, β-endorphin (Cowley et al., 2001); co-localize with cocaine- and CART (Lau et al., 2018)	Arcuate POMC neurons co-release α-MSH + β-endorphin + CART (Cowley et al., 2001); α-MSH → MC4R for anorexia; β-endorphin → MOR for rewards modulation – divergent co-transmission within single neurons (Mercer et al., 2013)
PACAP	PACAP-27, PACAP-38 (Sherwood et al., 2000)	ADCYAP1 (Sherwood et al., 2000)	PAC1R, VPAC1R, VPAC2R (Sherwood et al., 2000)	PACAP co-localizes with glutamate in the retinohypothalamic tract (Hannibal et al., 2000), with catecholamines in adrenal medulla and autonomic ganglia (Kuri et al., 2009)	Retinohypothalamic tract fibers co-release PACAP + glutamate onto SCN (Hannibal et al., 2000); glutamate mediates acute phase shift; PACAP provides sustained photic entrainment – functionally distinct roles at the same synapse (Hannibal et al., 2000)

Key peptides, biosynthetic precursor genes, receptor classes, patterns of coexistence with classical transmitters, and representative examples of co-transmission are listed. 5-HT, serotonin; ACh, acetylcholine; ACTH, adrenocorticotropic hormone; AM, adrenomedullin; ANP/ANF, atrial natriuretic peptide/factor; AVP, arginine vasopressin; BFCNs, basal forebrain cholinergic neurons; BNP, brain natriuretic peptide; CART, cocaine- and amphetamine-regulated transcript; CCK, cholecystokinin; CGRP, calcitonin gene-related peptide; CNP, C-type natriuretic peptide; CRH, corticotropin-releasing hormone; DA, dopamine; DNP, dendroaspis natriuretic peptide; DOR, δ-opioid receptor; DYN, dynorphin; GABA, γ-aminobutyric acid; GIP, glucose-dependent insulinotropic polypeptide; GLP, glucagon-like peptide; GnRH, gonadotropin-releasing hormone; GHRH, growth hormone-releasing hormone; KNDy, Kisspeptin, Neurokinin B, and Dynorphin; KOR, κ-opioid receptor; MC4R, melanocortin 4 receptor; MOR, μ-opioid receptor; MSH, melanocyte-stimulating hormone; MSNs, medium spiny neurons; NA, noradrenaline; NKA, neurokinin A; NKB, neurokinin B; NMJ, neuromuscular junction; NOP, nociceptin/orphanin FQ receptor; NPR-B, natriuretic peptide receptor B; NPTX2, neuronal pentraxin 2; NPY, neuropeptide Y; NT, neurotensin; OT, oxytocin; PACAP, pituitary adenylate cyclase-activating polypeptide; PDYN, prodynorphin; PENK, proenkephalin; PHI/PHM, peptide histidine isoleucine/methionine; POMC, pro-opiomelanocortin; PNOC, pronociceptin; PP, pancreatic polypeptide; PVN, paraventricular nucleus; PYY, peptide YY; SCN, suprachiasmatic nucleus; SP, substance P; SST, somatostatin; TRH, thyrotropin-releasing hormone; VIP, vasoactive intestinal peptide.

The discovery that neuropeptides coexist with classical low–molecular-weight neurotransmitters transformed our understanding of synaptic organization. Seminal neuroanatomical and immunocytochemical studies by Tomas Hökfelt and collaborators demonstrated that peptides are not confined to specialized neurosecretory cells but are widely distributed across central and peripheral neuronal populations (Hökfelt et al., 1980a,b; Lundberg et al., 1980; Lundberg and Hökfelt, 1983). These observations challenged the rigid interpretation of Dale’s principle. They introduced a new framework built on three related but distinct concepts: coexistence (multiple transmitters within a single neuron), costorage (multiple transmitters within a single vesicular compartment), and cotransmission (activity-dependent corelease).

**FIGURE 1:**
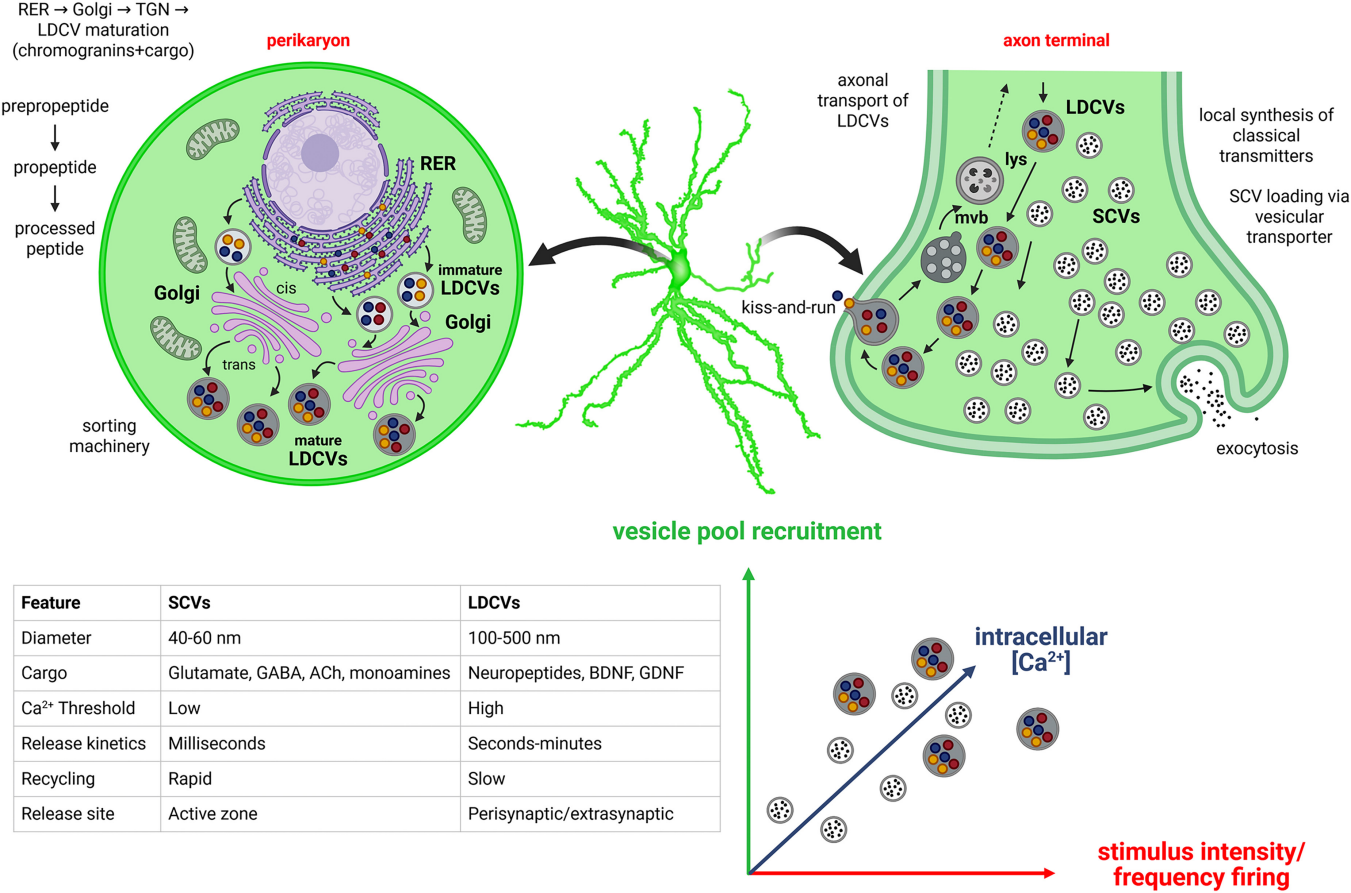
Dual vesicular architecture underlying cotransmission. Structural organization of classical neurotransmitter and neuropeptide storage within a single neuron. SCVs (∼40–60 nm) cluster at presynaptic active zones and contain classical neurotransmitters (e.g., glutamate, GABA, ACh), enabling rapid, Ca^2+^ nanodomain–dependent exocytosis. LDCVs (∼100–500 nm), containing neuropeptides and associated proteins (colored dots), are distributed throughout the presynaptic bouton and along axonal shafts, often positioned outside active zones. LDCVs require broader cytosolic Ca^2+^ elevations for release and exhibit slower recycling kinetics. Membrane remnants of exhausted and/or partly exhausted LDCVs are recycled by multivesicular bodies or late endosomes/lysosomes. These structures can then undergo retrograde transport back to the cell body via dynein-dependent mechanisms. Once returned to the soma, the material can be directed to lysosomes for degradation or potentially to the TGN for reprocessing. The spatial and molecular segregation of the two types of vesicles constitutes the structural basis for differential release and stimulus-dependent cotransmitter recruitment. ACh, acetylcholine; LDCVs, large dense core vesicles; mvb, multivesicular body; lys, late lysosome; RER, rough endoplasmic reticulum; SCVs, small clear vesicles; TGN, trans Golgi network. Partly created in BioRender (2026) https://BioRender.com/7jamx5s.

**FIGURE 2:**
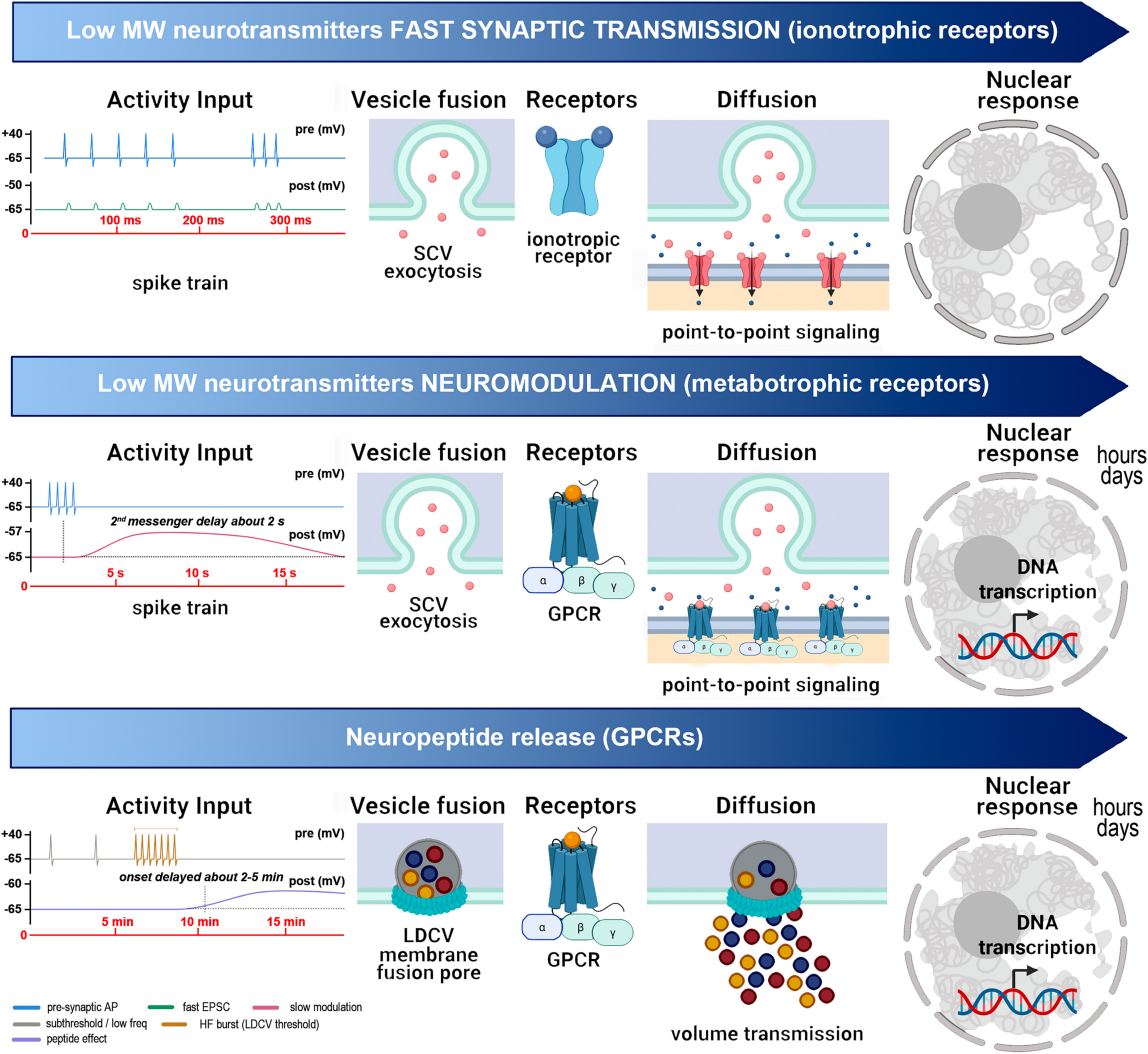
Activity-dependent temporal stratification of cotransmission. Frequency-dependent recruitment of classical neurotransmitters and neuropeptides. Low-frequency neuronal firing selectively triggers SCV exocytosis, thereby mediating fast synaptic transmission via ionotropic receptors on a millisecond timescale. At higher frequencies, metabotropic GPCRs can be activated, thereby modulating synaptic activity and, in some cases, leading to transcriptional activation. High frequency bursts produce global Ca^2+^ elevations sufficient to trigger LDCV exocytosis and neuropeptide release. Peptides diffuse beyond the synaptic cleft and activate GPCRs via volume transmission, generating slower modulatory effects over seconds to minutes and inducing transcriptional responses over longer timescales. This hierarchical activation links firing patterns to qualitative changes in chemical output. AP, action potential; EPSC, excitatory postsynaptic current; GPCR, G protein-coupled receptor; HF, high-frequency; LDVCs, large dense core vesicles; SCVs, small clear vesicles. Partly created in Biorender (2026) https://biorender.com/rr0juln.

The coexistence of different transmitters in a single neuron is now regarded as the rule rather than an exception. In most cases, coexistence concerns neuropeptides and classical transmitters. Neurons that synthesize neuropeptides and classical transmitters operate through two spatially and kinetically distinct vesicular systems (Merighi, 2002, 2018). Small clear vesicles (SCVs) store glutamate (Merighi et al., 1991), GABA (Merighi et al., 1989a), ACh (Brown, 2006), monoamines (Mulvihill, 2019), and purines (Burnstock, 1983, 2007), and primarily support ultrafast, point-to-point synaptic transmission. In contrast, large dense-core vesicles (LDCVs) contain neuropeptides (Salio et al., 2006) and, frequently, high-molecular-weight signaling proteins, such as certain neurotrophic factors (Salio et al., 2007), and require sustained or high-frequency stimulation for exocytosis. This molecular segregation is not incidental; it constitutes a regulatory architecture that enables stimulus–intensity–dependent recruitment of distinct chemical signals (Figures 1, 2). A further element of regulation lies in the possibility that both individual SCVs and LDCVs store more than a single messenger molecule. Costorage in LDCVs is widely accepted nowadays, whereas SCVs remain an open issue (Mondoloni and Mameli, 2022). Electrophysiological observations have demonstrated co-release of GABA and glutamate at basal ganglia-to-habenula synapses (Shabel et al., 2014; Kim et al., 2022). Yet, an ultrastructural study suggested that the two amino acid transmitters are stored in different SCVs based on the localization of the respective vesicular transporters (Root et al., 2018).

As a consequence of coexistence and/or costorage within a single neuron, cotransmission may occur. The existence of low- and high-molecular-weight neuromessengers introduces temporal coding into neural communication (Merighi et al., 1989b; Merighi, 2002, 2009, 2017). As mentioned, low-molecular-weight classical transmitters mediate fast synaptic transmission. The latter occurs after activation of ionotropic receptors with effects on the millisecond scale. Yet, these transmitters also act through metabotropic G-protein–coupled receptors (GPCRs) that signal on a slower time scale and often function as autoreceptors, providing negative feedback and thus modulating synaptic responses (Bowery et al., 2002; Fredholm et al., 2005; Bockaert et al., 2006; Gilsbach and Hein, 2008; Niswender and Conn, 2010; Beaulieu and Gainetdinov, 2011). High-molecular-weight neuropeptides primarily act through GPCRs (Vaudry and Seong, 2014), generate modulatory responses spanning seconds to minutes, and may initiate transcriptional programs lasting hours. In addition, some of the larger molecules that can be cargoed in LDCVs, i.e., brain-derived neurotrophic factor (BDNF) (Aoki et al., 2000) and glial-derived neurotrophic factor (GDNF) (Merighi, 2016; Ferrini et al., 2021), signal through receptor tyrosine kinases (RTKs). Thus, cotransmission represents a form of biochemical multiplexing, enabling a single neuron to encode both fast synaptic accuracy and slow network modulation without necessitating structural reconfiguration (Nusbaum et al., 2017; Svensson et al., 2019). Notably, recent research in *Drosophila* has demonstrated that posttranscriptional regulation of fast transmitter cotransmission also provides a mechanism for the reversible tuning of neuronal output (Chen et al., 2023). These authors have, in fact, demonstrated that many glutamatergic and GABAergic cells also transcribe cholinergic genes but fail to accumulate cholinergic effector proteins, and undergo transient translation of cholinergic proteins, indicating that suppression of cotransmission is actively modulated. Evolutionary data suggest that this arrangement is not superfluous but rather represents distinct signaling systems. In primitive metazoans, neuropeptides probably emerged before fast amino acid transmitters and functioned through diffuse modulatory networks (Jékely, 2021). Later in evolution, the emergence of rapid synaptic transmission increased the speed of pre-existing modulatory frameworks. More recent nervous systems, therefore, integrate ancient peptide-based communication with high-fidelity point-to-point transmission, preserving both flexibility and computational efficiency. Beyond its molecular elegance, coexistence carries profound functional consequences. Peptide–neurotransmitter interactions regulate synaptic plasticity, oscillatory dynamics, motivational states, endocrine rhythms, sensory gain control, and autonomic output (Baudry, 2002). However, dysregulation of cotransmission contributes to network instability, chronic pain, addiction, and stress-related disorders, to name a few examples. The dual-transmitter architecture is therefore not merely a biochemical curiosity but a central determinant of neural computation and adaptability.

This review synthesizes current knowledge of how neuropeptides and classical neurotransmitters are organized at the molecular level, how they are released, how they interact within systems, their evolutionary history, and their roles in disease. These features support the idea that cotransmission is a fundamental way the nervous system processes information, expanding its ability to encode it through multiple chemicals in both time and space.

## 2 Molecular architecture of dual transmitter systems

The coexistence of neuropeptides and classical neurotransmitters is not simply a matter of coexpression; it is part of a highly organized molecular architecture that segregates synthesis, packaging, trafficking, and release into parallel but interacting pathways. This dual-system organization provides the structural basis for activity-dependent chemical multiplexing.

### 2.1 Neuropeptide biosynthesis, LDCV biogenesis, and termination of peptidergic neurotransmission

The synthesis of neuropeptides involves a series of biochemical steps that begin with large precursor proteins. These prepropeptides are produced from specific genes and translated on the rough endoplasmic reticulum (Sossin and Scheller, 1991). After translation, a second step involves the formation of propeptides upon removal of the signal peptide. These propeptides then undergo several changes, including endoproteolytic cleavage, carboxypeptidase trimming, amidation, sulfation, and glycosylation (Seidah and Chrétien, 1992; Hook et al., 2008).

Unlike classical transmitters, which are produced locally in nerve terminals, transcription of neuropeptide mRNAs in the neuronal soma controls neuropeptide production in response to activity levels (Figure 1). Transcriptional control is mediated by cyclic AMP response element-binding protein (CREB) and c-Fos (Chowdhury et al., 2023). Increases in intracellular Ca^2+^ and cAMP activate these two transcription factors. Control of gene expression allows neurons to adjust their peptide content in response to conditions such as developmental stage, injury, stress, or prolonged network stimulation. Therefore, the plasticity of peptidergic neurons enables a broader functional scope of cotransmission. LDCV biogenesis requires careful cargo collection and sorting. The ultrastructure of mature LDCVs has been widely investigated using electron microscopy. Typically, LDCVs measure 100–500 nm in diameter and contain an electron-dense core composed of packed peptides and associated proteins (Merighi et al., 1989a). Proteins of the granin family contribute to the condensation of cargo inside LDCV, while additional conserved proteins–including the endosome-associated recycling protein (EARP) complex, EARP-interacting protein 1 (EIPR-1), and conserved coiled-coil protein 1 (CCCP-1)–participate in vesicle maturation and membrane trafficking (Winkler et al., 1991; Winkler and Fischer-Colbrie, 1998; Bartolomucci et al., 2011). Importantly, LDCVs frequently contain multiple peptides/high–molecular-weight signaling molecules. Ultrastructural investigations revealed that these costored messengers can be present in specific stoichiometric proportions, suggesting a coordinated packaging process rather than random aggregation (Salio et al., 2007). The question of whether different groups of LDCVs exhibit distinct peptide compositions remains under investigation, and answering it would be important for clarifying several functional aspects of LDCV biology. The movement of LDCVs from the cell body to axon terminals or dendrites depends on microtubules and is primarily mediated by kinesin-3 (KIF1A) (Hirokawa et al., 2010) and dynein–dynactin (Waterman-Storer et al., 1997) motor complexes. Whereas anterograde axonal transport depends on KIF1A, the existence of about 50% dendrites with mixed polarity in rodents (Tas et al., 2017) permits, in principle, both kinesin and dynein to carry LDCVs distally. Vesicle transport is bidirectional and dynamic, with periods of pausing and varying movement lengths (Kwinter et al., 2009). Because peptide replenishment requires synthesis in the cell body and long-distance transport, replacing them after they are released is relatively slow (Wong et al., 2012).

Peptidergic signaling is ended through several overlapping mechanisms. These mechanisms are much slower than those that terminate fast neurotransmission mediated by classical transmitters. The first and most immediate factor is the simple diffusion from the release site. Because neuropeptides are often released extrasynaptically and act via volume transmission (Zoli et al., 1999; Vizi et al., 2004; Fuxe and Borroto-Escuela, 2016), their concentration at target receptors decays as they diffuse through the extracellular space. Extracellular proteolytic degradation is the primary mechanism for terminating neuropeptide signals (Defea et al., 2000); other mechanisms involve internalization of the peptide or peptide-receptor complex, followed by intracellular degradation (Babilon et al., 2013). The key extracellular peptidases include neprilysin (neutral endopeptidase/enkephalinase, NEP), angiotensin-converting enzyme (ACE), dipeptidyl peptidase 4 (DPP4), aminopeptidases, and endopeptidase 24.15 (neurolysin).

Neutral endopeptidase/enkephalinase is the best-characterized neuropeptidase. It degrades substance P (SP) (Skidgel et al., 1984), as well as enkephalin (Roques et al., 1982), bradykinin (Campbell, 2018), calcitonin gene-related peptide (CGRP) (Krämer et al., 2009), neurotensin (NT) (Skidgel and Erdös, 2004), and OT (Riley et al., 1995), among others. ACE (Skidgel and Erdös, 2004), DPP4 (Canneva et al., 2015), aminopeptidases (Ramírez et al., 2011), and endopeptidase 24.15 (Dahms and Mentlein, 1992) also contribute in a peptide- and context-dependent manner. The bioactivity of neuropeptide Y (NPY) is either N-terminal modulation by DPP4-like enzymes with respect to receptor selectivity or proteolytic degradation by NEP or meprins, thereby abrogating signal transduction (Wagner et al., 2015). Enzymatic degradation via extracellular peptidases rather than by reuptake mechanisms results in cerebral half-lives for neuropeptides such as AVP and OT of approximately 20 min in cerebrospinal fluid (Mens et al., 1983), whereas CGRP has a half-life of 7 min in plasma (Kraenzlin et al., 1985), but there are no data in extracellular fluids. Finally, because most neuropeptide receptors are GPCRs, the signaling cascade may also be terminated at the receptor level. Termination of neuropeptide signaling at this level may occur through uncoupling of receptors from heterotrimeric G proteins and receptor endocytosis (Defea et al., 2000). Specifically, agonist-occupied receptors are phosphorylated by G-protein-coupled receptor kinases (GRKs), which recruit β-arrestins. Steric binding by β-arrestin interferes with further G protein coupling, leading to desensitization of G protein-dependent signaling (Grady et al., 1996; Marvizon et al., 1999). Notably, β-arrestin-dependent desensitization and internalization of receptors leads to suppression of GPCR signaling for several hours.

### 2.2 Classical neurotransmitter synthesis and small synaptic vesicle structure

Classical neurotransmitters–ACh, amino acids, monoamines, and purines–are synthesized locally within neuronal processes, mainly at presynaptic terminals. Their biosynthetic enzymes and vesicular transporters are advantageously positioned at these sites to ensure rapid supply and high-frequency release from 40 to 60 nm SCVs, which store these transmitters at high concentration (Fei and Krantz, 2009). Vesicular loading depends on dedicated transporters (e.g., vesicular glutamate transporters - VGLUTs - for glutamate, vesicular GABA transporter - VGAT - for GABA/glycine, vesicular monoamine transporter 2 - VMAT2 - for monoamines, vesicular acetylcholine transporter - VAChT - for ACh) driven by a proton electrochemical gradient (Onoa et al., 2010). SCVs are clustered at active zones and tethered near voltage-gated Ca^2+^ channels, ensuring nanodomain coupling between Ca^2+^ influx and vesicle fusion (Eggermann et al., 2011). The release of SCVs depends on the SNAP receptor (SNARE) complex. The complex comprises proteins, including vesicular synaptobrevins (v-SNAREs) and syntaxin/SNAP-25 (soluble NSF attachment protein 25) on target membranes (Südhof, 2008, 2012). Synaptotagmin primarily senses Ca^2+^. This molecular complex empowers exocytosis within milliseconds of depolarization. Importantly, SCVs are rapidly retrieved via clathrin-mediated endocytosis or ultrafast endocytic pathways, refilled, and recycled to terminate fast signaling (Watanabe et al., 2014). This very efficient cycle supports continued high-frequency transmission without requiring transcriptional upregulation. The tight spatial localization of SCVs to active zones safeguards point-to-point synaptic specificity. Classical transmitters therefore operate with high temporal and spatial precision, providing the fast computational backbone of neural circuits.

### 2.3 Vesicular segregation as a regulatory principle

The isolation of neuropeptides and classical transmitters into distinct vesicle classes is not redundant–it is mechanistically strategic. The core understanding is that the physical separation of neuropeptides into LDCVs and classical transmitters into SCVs is not an evolutionary accident or mere anatomical tidiness – it encodes distinct release rules, spatial ranges, and computational functions that would be mutually incompatible if co-packaged. Vesicular separation is mechanistically strategic because the vesicle type simultaneously determines three separable yet interacting properties: release threshold, spatial geometry, and downstream receptor coupling. SCVs and LDCVs, in fact, not only differ in size and ultrastructure, cargo composition, but also in Ca^2+^ sensitivity, release kinetics, recycling capacity, and spatial release domains. In addition, SCV fusion requires a high concentration of Ca^2+^ in microdomains near voltage-operated channels (Parekh, 2008). On the other hand, LDCV exocytosis normally requires higher and more sustained intracellular Ca^2+^ elevations, as well as repetitive or high-frequency stimulation (Cifuentes et al., 2008; Ludwig and Stern, 2015). Consequently, peptide release typically occurs after classical transmission has already initiated. As mentioned, LDCVs are not only located at active zones. Therefore, these vesicles can be released via exocytosis at either perisynaptic or extrasynaptic sites, thereby facilitating volume transmission (Zoli et al., 1999; Vizi et al., 2004; Fuxe and Borroto-Escuela, 2016). Thus, the capacity of LDCVs to be distributed across various neuronal compartments and released from multiple sites, rather than being confined to synapses, allows neuropeptides to exert influence on a broader range of cells, including those located at significant distances from the initial release site. Furthermore, the disparate recycling rates of these two messenger types contribute to their divergence (Eiden et al., 2022; Hevesi et al., 2025). Whereas SCVs are rapidly endocytosed for recycling at terminals, LDCVs are not recycled following exocytosis; thus, replenishing the LDCV pool requires *de novo* synthesis and transport of their contents. Therefore, peptide signaling is discontinuous and state-dependent rather than continuously sustainable. Together, these properties create a graded signaling architecture in which neurons can operate both fast synaptic transmission for precision and slow modulatory transmission for context integration. Vesicular segregation, therefore, acts as a built-in regulatory mechanism that converts stimulus intensity and duration into chemically distinct outputs (Cárdenas and Marengo, 2016). Mixing vesicle cargoes would not merely blur the signals – it would destroy the frequency-dependent, spatially graded, timescale-separated logic that makes co-transmitting neurons capable of encoding both fast information and slow states. Two examples of the strategic importance of vesicle separation are the co-release of NPY and GABA in hippocampal interneurons (van den Pol, 2012) and SP and glutamate in dorsal horn nociceptive circuits (De Koninck and Henry, 1991; Woolf and Salter, 2000).

In certain GABAergic hippocampal interneurons (basket cells, Schaffer collateral-associated cells), GABA is localized in SCVs and NPY in LDCVs. The strategic separation here operates at the level of stimulation frequency. A single action potential or a low-frequency train (<5 Hz) raises intraterminal Ca^2+^ to ∼1–10 μM at the active zone, which is sufficient to trigger SNARE-mediated fusion of docked SSVs via synaptotagmin-1. GABA is released with sub-millisecond latency into the synaptic cleft, activates postsynaptic GABA_*A*_ receptors, and produces rapid Cl^–^-mediated inhibition. LDCVs, by contrast, are physically excluded from the active zone. They are located ≥100–300 nm away, where local Ca^2+^ concentrations never reach the levels required for fusion machinery, which consists of synaptotagmin-7 or -9. Only during high-frequency burst firing (≥10–20 Hz, as occurs during theta oscillations or intense sensory drive) does global Ca^2+^ rise sufficiently across the terminal to trigger LCDV exocytosis. NPY is then released extrasynaptically, diffuses by volume transmission over hundreds of μm, and acts via G_i_-coupled Y_1_/Y_2_ receptors to suppress adenylyl cyclase, reduce presynaptic Ca^2+^ currents, and hyperpolarize postsynaptic neurons via G protein-coupled inward rectifier K^+^ (GIRK) channels. The strategic consequence is a two-layer inhibitory system with intrinsic frequency-detection: basal activity is handled by GABA alone (fast, local, restorable); pathological hyperactivity recruits NPY as a second, longer-lasting, spatially broader brake, providing a form of gain control that SCV-based signaling alone could never achieve. Merging NPY into SSVs would strip it of this frequency gating and eliminate the computational distinction.

Primary afferent C-fibers entering the dorsal horn of the spinal cord co-contain glutamate in SCVs and SP + CGRP in LDCVs. This pairing is the canonical model for the distinction between acute and persistent pain signaling. With innocuous or mild noxious stimulation, C-fibers fire at low frequency. SCV-glutamate release activates α-amino-3-hydroxy-5-methyl-4-isoxazolepropionic acid (AMPA) receptors on projection neurons, producing fast EPSPs that encode stimulus intensity in real time, thereby giving rise to the acute, sharp, well-localized component of pain. The Mg^2+^ block keeps NMDA receptors silent. During intense, sustained, or repeated noxious stimulation (high-frequency C-fiber burst), LDCVs are recruited. SP is released from extrasynaptic sites and binds NK1 receptors (NK1R - G_q_-coupled) on postsynaptic neurons and on astrocytes. NK1R activation raises Ca^2+^ via inositol triphosphate (IP3), activates protein kinase C (PKC), which phosphorylates and relieves Mg^2+^ block of NMDA receptors. This triggers a phenomenon called wind-up, with progressive amplification of postsynaptic responses that leads to central sensitization. CGRP simultaneously dilates blood vessels and activates glia. The entire mechanism is mechanistically strategic because the two signals serve qualitatively different computational roles on different timescales: glutamate/AMPA encodes acute pain; SP/NK1R encodes long-lasting pain and reconfigures the circuit for heightened sensitivity. Co-packaging these messengers in SCVs would either fire SP at every stimulus (thereby destroying its threshold meaning) or require a separate release mechanism for each (impossible within a single vesicle class).

### 2.4 Is the SCV/LDCV dicothomy absolute?

Co-storage of low-molecular-weight transmitters and neuropeptides within the same SCV is poorly established and considered atypical. In human sympathetic neurons of the lumbar ganglia, an electron microscopic study reported the concurrent presence of somatostatin (SOM) and NA (as detected by tyrosine hydroxylase immunostaining) in 40–60 nm vesicles (Järvi et al., 1987). However, we did not find other data in the literature supporting this observation. Conversely, LDCVs can store a low-molecular-weight neurotransmitter together with a neuropeptide. In sympathetic nerve homogenates and after immunocytochemical localization, NA was found to be stored in both SCVs and LDCVs, the latter of which also contain NPY (Fried et al., 1986; De Potter et al., 1987) or enkephalin (Fried et al., 1986; De Potter et al., 1987). These observations are well-documented and constitute the strongest example of the co-storage of a low-molecular-weight neurotransmitter and a neuropeptide within a single LDCV. Thus, the dichotomy between SCVs and LDCVs in terms of neurotransmitter content is not absolute, but the above observations likely represent remarkable exceptions to a general rule.

### 2.5 Costorage and molecular complexity of LDCVs

The possibility that several neuropeptides are costored in individual LDCVs adds further complexity to our understanding of neuropeptide signaling. Numerous peptides within a single LDCV can be released concurrently; however, evidence indicates that selective or partial release can occur via mechanisms such as kiss-and-run fusion (Artalejo et al., 1998; Rutter and Tsuboi, 2004; Zhang et al., 2011). The occurrence of flexible peptide release from the same vesicle under physiological and/or pathological conditions remains controversial. Peptides and neurotrophic factors coexist within LDCVs of a subset of primary afferent neurons (Merighi et al., 1989b; Merighi, 2002; Salio et al., 2007). These neurons give rise to C-fibers projecting to the spinal cord lamina II (Uenoyama et al., 2021). These colocalized molecules integrate rapid glutamate transmission (the main neurotransmitter of the C-fibers) with sustained modulation, contributing to central sensitization in chronic pain states (Merighi et al., 2008; Uenoyama et al., 2021). The possibility of distinct LDCV subtypes with distinct neurochemical profiles within the same neuron remains an open question. Advanced imaging, single-vesicle proteomics, and super-resolution microscopy may clarify whether vesicular identity indeed occurs and encodes functional specialization.

The dual-transmitter neuron is therefore built upon two partially independent but convergent secretory systems, a fast, locally recyclable synaptic machinery optimized for speed and fidelity, and a slower, gene-regulated system optimized for modulatory scope and plasticity. This architectural arrangement provides the mechanistic foundation for the temporal stratification and operational models of cotransmission discussed in the following section (Hnasko and Edwards, 2012).

## 3 Release dynamics and temporal stratification: from synaptic precision to network modulation

The previously discussed dual vesicular architecture underpins cotransmission. Its functional significance is apparent in release dynamics, wherein fluctuations in Ca^2+^ sensitivity, vesicle positioning, and recycling kinetics convert stimulus patterns into chemically stratified outputs. Consequently, neurons function as activity-dependent multiplexers, converting firing frequency and duration into qualitatively distinct signaling regimes.

### 3.1 Differential Ca^2+^ thresholds and vesicle recruitment

As mentioned, SCVs are closely associated with voltage-gated Ca^2+^ channels at active zones, where localized Ca^2+^ increases initiate rapid exocytosis. A single action potential usually triggers fusion, thereby facilitating synaptic transmission on the millisecond timescale (Medeiros et al., 2024). Conversely, LDCVs are frequently located away from active zones, requiring greater cytosolic Ca^2+^ increases for their exocytosis (Voets et al., 2001). The likelihood of their fusion is augmented by high-frequency firing, burst activity, extended depolarization, metabolic stress, and neuromodulatory priming (van den Pol, 2012). Because LDCV release depends on global Ca^2+^ accumulation rather than nanodomain coupling, peptide exocytosis typically occurs after classical transmitter release. This temporal sequence establishes a stimulus-intensity–dependent hierarchy: low activity elicits fast synaptic transmission alone, whereas sustained activity recruits peptidergic signaling. This graded recruitment is evident in multiple systems, including sympathetic neurons exhibiting adenosine 5′-triphosphate (ATP) → NA → NPY triphasic output (Cannon et al., 1986; Svensson et al., 2019), and primary sensory afferents releasing glutamate at low frequency but co-releasing SP and CGRP during persistent stimulation (Latremoliere and Woolf, 2009). Therefore, the movement of Ca^2+^ ions is pivotal to convert electrical patterns into layered chemical signals.

### 3.2 Spatial separation: point-to-point versus volume transmission

Classical neurotransmitters usually act on receptors located on nearby post- or presynaptic membranes. At high local concentrations, they activate post- or pre-synaptic receptors. These receptors respond quickly but are rapidly inactivated by desensitization, internalization (endocytosis), or antagonist blockade (Brodal, 2016). Therefore, the geometry and molecular assembly of the synapse enforce spatial specificity and limit diffusion (Freche et al., 2011). Neuropeptides released from LDCVs frequently act extrasynaptically. Released peptides can diffuse over tens to hundreds of micrometers, affecting groups of neurons beyond the immediate synaptic connection. This process mainly activates GPCRs located near or distant from the bouton of release (Özçete et al., 2024). This process–often termed volume transmission–allows state-dependent modulation of circuit excitability, synaptic plasticity thresholds, and oscillatory coherence. Importantly, spatial divergence does not imply functional independence. Peptides often also influence the synapses from which they are released. They act presynaptically, controlling Ca^2+^ influx into the terminal, or postsynaptically, modulating receptor phosphorylation (Fröhlich, 2016). Consequently, volume transmission can also exert a feedback effect on point-to-point signaling, thereby altering circuit gain.

### 3.3 Chronological layering of neural signaling

Coexistence of classical transmitters in SCVs and neuropeptides in LDCVs endows individual synapses with a frequency-gated, dual-timescale output that integrates phasic ionotropic signaling with sustained metabotropic modulation. The fast (milliseconds) response occurs after ionotropic receptor activation, leading to rapid depolarization or inhibition. This initial response encodes precise spike timing and synaptic integration (Bucher and Goaillard, 2011). A second temporally intermediate response follows (seconds to minutes) during which peptide-activated GPCRs influence ion channel activity, the release of intracellular Ca^2+^, kinase cascades, and an array of metabolic processes depending on the type of peptide and the context of activation (Lohse et al., 2014; Culhane et al., 2022). As a consequence, excitability, short-term plasticity, and network synchrony are regulated. Finally, sustained peptide signaling, which occurs over minutes to hours, can activate transcription factors like CREB (Montminy et al., 1990). Transcription factor activation, in turn, causes changes in gene expression that alter synaptic structure and the types of receptors expressed at synapses. This third temporal layer links transient activity to long-term alterations in brain function. Therefore, the timing of cotransmission extends a neuron’s influence beyond immediate electrical signals, leading to lasting structural changes (Vaaga et al., 2014; Monday et al., 2018). Remarkably, within this general framework, peptides are not redundant and convey information about the activity’s strength and duration.

### 3.4 Neuropeptide GPCRs

**FIGURE 3:**
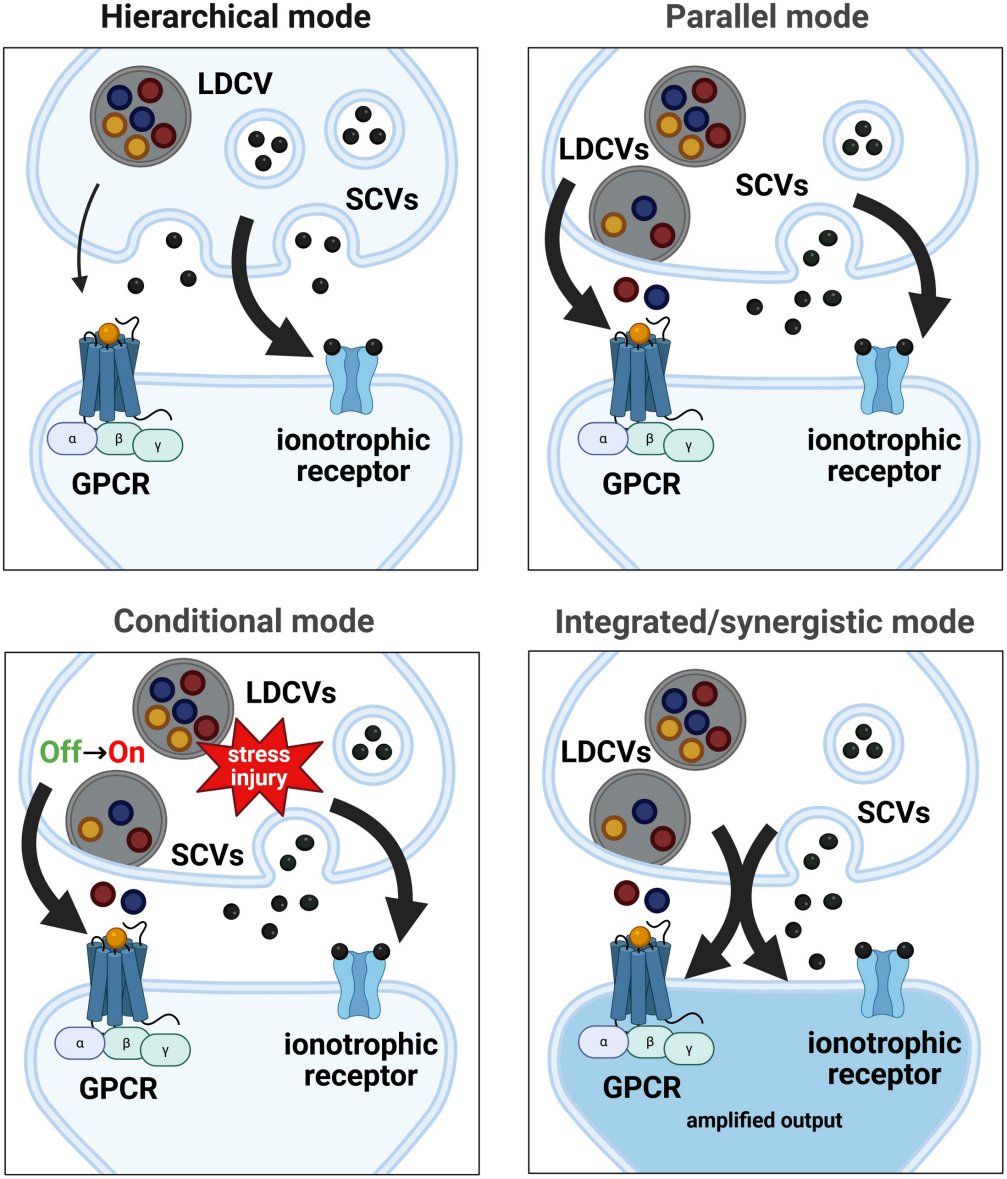
Operational models of neuropeptide–neurotransmitter cotransmission. In the hierarchical model, classical neurotransmission provides primary signaling, while peptides exert modulatory control at higher activity thresholds. In the parallel mode, classical transmitters and peptides act simultaneously on distinct receptor systems, producing complementary effects. In the conditional model, peptide release is activated only under specific physiological or pathological conditions (e.g., stress or injury). In the integrated/synergistic model, peptides and classical transmitters interact at pre- and postsynaptic levels to produce non-linear amplification or gating of circuit responses. These models are not mutually exclusive and may coexist within the same neuron, depending on the neuron’s activity state and circuit context. Created in BioRender (2026) https://BioRender.com/jjzzwvc.

As mentioned, neuropeptides act by activating specific cell-membrane receptors that are members of the GPCR superfamily, thereby stimulating downstream protein kinase signaling pathways and ultimately altering gene expression. The vast majority of the neuropeptides described to date act through GPCRs (Burbach, 2010). Table 2 lists the main types of GCPRs activated by the most widely investigated neuropeptides. It is worth noting that peptide GPCRs are not only expressed postsynaptically (as depicted for simplicity in Figure 3). Presynaptic autoreceptors are activated by the same neuropeptide produced in the nerve terminal, thereby regulating intrasynaptic feedback and influencing neurotransmission at adjacent synapses. G protein-coupled autoreceptors on both pre- and postsynaptic cells protect against overstimulation by releasing G protein βγ subunits, which activate GIRK channels postsynaptically and block voltage-activated calcium channels presynaptically. The Gβγ subunits can directly engage with SNARE exocytotic machinery to inhibit release (Betke et al., 2012).

**TABLE 2:** Main types of GPCRs activated by neuropeptides.

Neuropeptides	GPCRs	G protein	Cellular mechanisms	Main functions	References
Opioid peptides	μ, δ, κ Class A GPCRs	G_*i*_/_*o*_ (inhibitory)	Receptors transduce extracellular messages using Gα and Gβγ subunits, MAPK, and arrestin signaling pathways	CNS: Pain, mood, rewards mechanisms PNS: modulation of nociception, peripheral algesia.	Reeves et al., 2022; Dahliwal and Gupta, 2023
Tachykinins (SP, NKA)	NK1 Class A GPCR	G_q_/G_s_ (activatory)	SP increases both IP and cAMP, second messengers downstream of G_*q*_- and G_*s*_-mediated signaling pathways, respectively. SP released from primary afferent fibers during inflammation upregulates NK1 receptors in dorsal horn neurons, which control the expression of cytokines, chemokines, and transcription factors such as NF-κB. NKA signals potently via G_q_ but has reduced G_*s*_-stimulatory activity.	CNS: Pain, mood, nausea PNS: Gut motility, inflammation (skin, gastrointestinal tract, respiratory tract)	Martinez et al., 2015; Lang et al., 2020; De Zoysa et al., 2026; Zhang et al., 2026
CGRP	CLR/RAMP1 heterodimer Class B GPCR	G_s_ (activatory)	Dimerization of CLR with RAMP1 shows the highest affinity for CGRP	CNS: Central sensitization PNS: Vasodilation, migraine	Hay and Walker, 2017; Gingell et al., 2019
NPY, PYY, PP	Y1, Y2, Y4, Y5, and Y6 Class A GPCRs	G_*i*_/_*o*_ (inhibitory)	Regulation of adenylyl cyclase, activation of MAPK, regulation of intracellular Ca^2+^ concentrations, and activation of G protein-coupled GIRK channels	CNS: Anxiety, feeding, cognition. Y_1_ receptors modulate cell activity postsynaptically, whereas Y2 receptors function presynaptically as autoreceptors and heteroreceptors, regulating NPY and co-expressed neurotransmitter release. PNS: Vasoconstriction, sympathetic tone, pain. N-terminal cleaved NPY variants primarily mediate angiogenesis and neurotransmitter release inhibition through Y_2_.	Marcos and Coveñas, 2022; Nelson et al., 2024; Singanwad et al., 2025
VIP PACAP family	VPAC1, VPAC2 (bind both peptides) PAC1 (only binds PACAP) Class B GPCRs	G_s_ (activatory), G_q_ (activatory), or G_*i*_ (inhibitory)	Primarily coupled to adenylate cyclase via G_s_. Other pathways are activated via βγ subunits and by alternative coupling to G_*i*_ and G_q_.	CNS: Circadian rhythm, neuroprotection PNS: Immune modulation, gut motility	Hirabayashi et al., 2018; Tasma and Hay, 2025

cAMP, cyclic adenosine monophosphate; CGRP, calcitonin gene-related peptide; CLR, calcitonin receptor-like receptor; CNS, central nervous system; GIRK, G protein-coupled inwardly rectifying potassium; GPCRs, G protein coupled receptors; IP, inositol phosphate; MAPK, mitogen-activated protein kinase; NF-κB, nuclear factor kappa-light-chain-enhancer of activated B cells; NKA, neurokinin A; NPY, neuropeptide Y; PAC1, pituitary adenylate cyclase-activating polypeptide type I receptor; PACAP, pituitary adenylate cyclase-activating polypeptide; PNS, peripheral nervous system; PP, pancreatic polypeptide; PYY, Peptide YY; RAMP1, receptor activity modifying protein 1; SP, substance P; VIP, vasointestinal polipeptide; VPAC, vasoactive intestinal peptide receptors; Y, NPY receptors.

### 3.5 Operational models of cotransmission

Based on temporal dynamics and receptor expression, four operational models can be described (Figure 3).

In the so-called hierarchical model, classical transmitters are the main signaling molecules (Svensson et al., 2019). They mediate fast point-to-point signaling upon the arrival of every action potential. Neuropeptides serve as amplifiers or stabilizers of ionotropic transmission, being released from LDCVs exclusively under conditions of intense or repetitive presynaptic activity above a firing-rate threshold that cannot be met by SCV-mediated signaling alone (Vaaga et al., 2014). The main limitation of this model is its unidirectionality, i.e., it does not account for cases in which peptide signaling preconditions or gates the classical transmitter’s efficacy, nor for tonic peptide release. This pattern is often seen in circuits that respond to stressful situations, such as nociceptive circuits (Liu and Sandkühler, 1995), where antibody microprobe measurements in the dorsal horn showed that SP appears extracellularly only during high-intensity nociceptive bursts, whereas glutamate signals are present at every stimulus (Duggan and Furmidge, 1994). Similarly, in sympathetic fibers, low-frequency stimulation releases only NA, whereas high-frequency bursts additionally release NPY, further increasing vasoconstriction (Lundberg and Hökfelt, 1983). As a last example, in cholecystokinin (CCK) immunoreactive basket cells, GABA release is asynchronous throughout, but CCK modulation of network oscillations emerges only during sustained interneuron recruitment (Hefft and Jonas, 2005). According to this model, traditional neurotransmitters and neuropeptides may also affect different domains within a single neuron (e.g., the axon and dendrites) or across different neurons and neuron types (Neuhofer and Kalivas, 2023). In hypothalamic neurons, peptide release from dendrites can occur independently of the more conventional axonal transmission, leading to differences in how signals are sent, depending on the state of neuron activation (Landry et al., 2003; Ludwig et al., 2016).

In parallel mode, co-released messengers enable a single neuron to address two distinct downstream circuits simultaneously by acting on the same cell that expresses different receptor types, or on separate cell populations that express different receptors. The two signals don’t have to necessarily interact. Bona fide, pure parallelism is most likely uncommon. A certain amount of local crosstalk is practically inevitable because LDCVs and SSVs are often released from the same terminal, making it likely that messengers interact in accordance with the hierarchical or integrated models. Yet, three examples of this type of interaction are given below. Spinal cord motor neurons co-release ACh and CGRP; the former acts on skeletal muscle nicotinic receptors and the latter on vascular smooth muscle CGRP receptors (Zaidi et al., 1987). In this case, the two messengers act on completely separate effector systems, with no cross-talk. Yet CGRP positively modulates synaptic transmission at the neuromuscular junction by locally inhibiting acetylcholinesterase expression (Rossi et al., 2003). In the crustacean stomatogastric ganglion, proctolin, an arthropod neuropeptide, and GABA from the same neuron activate entirely different postsynaptic cells, reconfiguring distinct circuit elements in parallel (Nusbaum and Blitz, 2012). Hypothalamic agouti-related peptide/protein (AgRP) neurons co-release GABA, AgRP, and NPY onto three distinct types of receptors, each expressed on non-overlapping target neuronal populations in the arcuate nucleus–paraventricular nucleus circuit (Andermann and Lowell, 2017).

In the conditional model, peptide release is activated only under specific physiological or pathological conditions, such as metabolic state, hormonal status, stress, and disease. As a limitation of this model, one has to consider that the mechanistic linkage between the state variable (e.g., a circulating hormone) and the LDCV release machinery is often only partially characterized. It is unclear in many systems whether state modifies release probability *per se*, vesicle pool composition, or postsynaptic receptor expression, as these mechanisms produce identical phenomenology but rely on very different cellular events. As an invertebrate example, in *C. elegans*, tyraminergic Rab3-interacting molecules (RIM) neurons co-release tyramine (a small neurotransmitter analog) and FMRFamide-like peptide 18 (FLP-18) (a neuropeptide) in response to mechanical stress. The tyramine immediately suppresses head movement, while the FLP-18 acts on a longer timescale through the natriuretic peptide receptor 5 (NPR-5) to enhance turning and sustain high locomotion speed (Florman and Alkema, 2022). There are several examples to support this model in mammals. They include the dramatic increase in OT release during parturition and lactation as a consequence of hormonal state-dependence, while basal glutamate co-transmission is unaffected, and dendritic peptide release is conditionally enabled by the retraction of glial sheaths (Theodosis et al., 1986a,b); the upregulation of corticotropin-releasing hormone (CRH)/NPY corelease ratio from central amygdala neurons under chronic stress (McGuire et al., 2011); the fasting-dependent selective amplification of NPY release from arcuate neurons without proportional changes in GABA co-transmission that demonstrates an energy-status gating (Tong et al., 2008); and the shift in vagal CCK peptide tone in relation to the gut hormonal state, conditionally enabling or suppressing satiety circuit signaling by ghrelin and leptin (Chung and Heldsinger, 2011).

According to the integrated or synergistic model, peptides and classical transmitters converge on common intracellular pathways, interacting at pre- and postsynaptic levels to produce non-linear amplification or gating of circuit responses. Interaction may occur at the level of second-messenger cascades, receptor heteromerization, or network feedback to produce nascent computational properties that each signal could not generate alone. Because it necessitates the independent regulation of each co-released signal *in vivo* at physiological concentrations and timescales, interaction is hard to verify scientifically. Many purported synergies could in fact be the result of supraphysiological peptide application-related experimental artifacts. There are several examples of this type of interaction. The co-release of neuropeptides by dopaminergic neurons in the basal ganglia is an example of this integrated type of action, where modulatory interactions alter both motor function and rewards processing (Rice et al., 2011). In the limbic system, the coexistence of glutamate and dynorphin (DYN) is a key functional feature. DYN is often co-released to act as a presynaptic brake, inhibiting further glutamate release and creating a gating mechanism that prevents excitotoxicity and modulates synaptic plasticity processes necessary for learning (Wagner et al., 1993). In the ganglia of the autonomic nervous system (ANS), ACh and luteinizing hormone-releasing hormone (LHRH) provide a classic example of a non-linear temporal response arising from synergistic interactions between the two molecules. Namely, ACh produces a rapid, brief action potential, whereas LHRH generates a slow depolarization that lasts for minutes (Jan and Jan, 1983; Lundberg and Hökfelt, 1983). As a consequence, the neuron shifts from a short-lasting response to sustained excitability. In the cortex and amygdala, GABA is the main inhibitory neurotransmitter, but its effect can be brief. NPY enhances GABAergic signaling at the postsynaptic level and reduces excitatory neurotransmitter release at the presynaptic level (Bacci et al., 2002; Molosh et al., 2013). Together, these molecules act as a powerful anti-stress system, “switching off” anxiety-related circuits much more effectively than GABA alone. In mesolimbic rewards areas, NT modulates dopamine signaling by altering the affinity of D2 dopamine receptors. In the presence of the peptide, the dopamine response is amplified, making a stimulus significantly more behaviorally salient (von Euler et al., 1989; Piccart et al., 2015).

According to most available data, these four models represent context-dependent operating modes of the same cotransmission apparatus rather than mutually exclusive alternatives. There are indeed the following unifying variables in support of such an interpretation: the LDCV release probability depends on the firing pattern (burst vs. sparse), vesicle pool composition and postsynaptic receptor sensitivity are determined by the physiological state, signals converge or diverge depending on the target identity, and the same neuron can multiplex over timescales due to the difference between slow peptide volume transmission and quick synaptic transmission. As Nusbaum et al. (2017) clearly discuss and exemplify, cotransmission systems are highly dynamic and best understood when one keeps in mind that the neuron does not use a fixed signaling strategy but instead chooses a combination most appropriate to current computational demand.

### 3.6 Frequency coding as chemical coding

Without changing anatomical connectivity, peptide recruitment increases computational dimensionality. Neurons, therefore, encode information not only in spike timing but also in the molecular composition of their output messengers (Paul et al., 2017). Such chemical multiplexing may enhance robustness under sustained stimulation, network stability during oscillatory activity, plasticity induction thresholds, and homeostatic compensation (Kundu et al., 2019). However, it also introduces vulnerability: dysregulated peptide release can shift circuit set points, contributing to chronic pain, addiction, epilepsy, or stress-related disorders. In brief, release dynamics convert the dual vesicular architecture into a functional hierarchy of signaling layers. Differences in Ca^2+^ sensitivity, spatial diffusion, receptor kinetics, and recycling capacity generate stimulus-dependent chemical stratification. Cotransmission thus represents a scalable regulatory system that integrates synaptic precision with network modulation and long-term plasticity. Examining how this layered signaling system evolved and is structured across species is the next obvious step toward better understanding how it operates.

## 4 Evolutionary origins of cotransmission

Cotransmission is not a late specialization of complex brains, but rather a well-preserved characteristic of neural systems. According to comparative neurobiology, peptide-based signaling predates fast synaptic transmission, and modern dual-transmitter neurons represent evolutionary layering rather than redundancy. The cohabitation of neuropeptides and classical transmitters thus constitutes an accretive design principle driven by rising needs for speed, precision, and computing scalability.

### 4.1 Peptides: ancestral signaling molecules

Neuropeptides are among the oldest intercellular mediators among metazoans (Jékely, 2021). Peptidergic signaling pathways are present in primitive species such as cnidarians (Grimmelikhuijzen and Westfall, 1995). It is well established that neuropeptides are the main class of transmitters in these animals, but there is evidence that classical low-molecular-weight neurotransmitters are also present in these species. Evidence is moderate-strong for glutamate, moderate for ACh and 5-HT, weak for GABA and catecholamines (Kass-Simon and Scappaticci, 2004; Anctil, 2009; Faltine-Gonzalez and Layden, 2019). Based on comparative genomics and neurochemistry, small-molecule neurotransmitters arose after neuropeptide-based signaling, probably coinciding with the emergence of bilaterians about 550–600 million years ago. Glutamatergic and GABAergic systems originated from amino acid metabolism that predates their neurotransmitter roles; their co-option as fast-acting signaling molecules appears to be a bilaterian innovation through convergent evolution (Moroz and Kohn, 2016). Current evidence supports an independent, parallel origin of small-molecular transmitters rather than derivation from neuropeptides, which are considered the more ancestral and indispensable signaling system. In accordance with this line of thought, it is highly unlikely the existence of organisms with only small molecular transmitter systems and no neuropeptides.

Cnidarians have neural networks but lack the complex synaptic mechanisms found in bilaterians. Peptides in these species regulate muscle contractions, feeding, reproduction, and rhythmic behaviors, primarily via diffuse, volume-based communication. Also, the evolutionary age of peptide GPCRs, many of which share similar forms across different animal species, implies that neuromodulatory signaling predated fast ionotropic transmission (Svensson et al., 2019). In early neural systems, peptides most likely functioned as idle integrators of physiological states rather than mediators of rapid responses to internal and/or external changes (Kupfermann and Kandel, 1970; Lloyd et al., 1987; Nassel, 2002; Nässel and Winther, 2010; Balog et al., 2012; Quiroga et al., 2015; Getz et al., 2020; Kreshchenko et al., 2021). This fundamental modulatory function is still present in contemporary brain circuitry. Even in vertebrates’ highly specialized brains, peptidergic transmission exhibits widespread, state-dependent control, implying that it has evolved rather than been replaced.

### 4.2 The advent of fast synaptic transmission

The emergence of classical low-molecular-weight neurotransmitters, e.g., glutamate and GABA, which use fast, point-to-point signaling primarily via ligand-gated ion channels, provided a novel means of communication in evolution (Burkhardt and Jékely, 2021). The emergence of specific presynaptic active zones and densely packed postsynaptic densities facilitated millisecond precision and spike-to-spike transmission. Rather than displacing peptidergic systems, rapid synaptic transmission was added to existing modulatory networks (Jékely, 2021). This dual-mode system enabled neural circuits to provide both immediate automatic responses and long-term contextual modification. From an evolutionary perspective, cotransmission may represent the merger of two temporally divergent communication strategies: the ancient, slow, diffuse peptidergic signaling and the more recent, quick, synaptically limited classical transmission. The conservation of both systems in evolution suggests a selective benefit in maintaining chemical variation within individual neurons.

### 4.3 Cotransmission in invertebrates

Research on invertebrates has helped improve understanding of cotransmission at a fundamental level. Neurons in the mollusk *Aplysia californica* co-release peptides and classical transmitters that modulate synaptic strength and result in behavioral plasticity (Kupfermann and Kandel, 1970; Lloyd et al., 1987). In this species, peptide cotransmission is crucial for sensitization and habituation of neural networks, revealing the early integration of fast and slow signaling in learning. Similarly, in the arthropod *Drosophila melanogaster*, multiple neurons express neuropeptides in addition to ACh, glutamate, or GABA. Peptides govern circadian cycles, eating behavior, and stress responses, and they often act across larger cellular domains than classical transmitters (Nassel, 2002; Nässel and Winther, 2010; Venken et al., 2011). Invertebrate data show that cotransmission is not a vertebrate innovation, but rather a general organizational technique. In these simpler creatures, cotransmission typically takes the form of a modulatory intersection: classical transmitters encode individual synaptic events, while peptides regulate circuit gain, excitability thresholds, and behavioral state.

### 4.4 Vertebrate expansion and functional diversity

With the evolution of vertebrates, the variety of peptides and receptor subtypes increased substantially (Burbach, 2010, 2011). Today, about 70 neuropeptide-encoding genes have been identified. The genes encoding classical neuropeptides are grouped into at least 18 families based on precursor and peptide structure (Burbach, 2011). Gene duplication events expanded the repertoire of neuropeptide precursors and GPCR families, enabling greater modulatory specialization. Cotransmission occurs across almost all major transmitter systems in animals (de Mendoza et al., 2014; Sebé-Pedrós et al., 2018). Glutamatergic neurons may coexpress neuropeptides such as SP or neurotrophins (Merighi et al., 1991; Martin and Finsterwald, 2011); GABAergic interneurons may contain SOM (Watt and Florack, 1993), vasoactive intestinal peptide (VIP) (Brecha et al., 1984), or CCK (Freund and Katona, 2007); monoaminergic neurons can corelease DYNs, NT, or other modulators (Maejima et al., 2013); and autonomic neurons generate a triphasic output comprising ATP, NA, and peptides (Macarthur et al., 2011). Diversification points to evolutionary refinement rather than redundancy. As circuit complexity rose, cotransmission enabled single neurons to encode multidimensional information while preserving anatomical connectedness.

### 4.5 Evolutionary logic of chemical multiplexing

From an evolutionary standpoint, cotransmission has proven highly advantageous (Vaaga et al., 2014; Svensson et al., 2019; Moroz and Romanova, 2021). Cotransmission enhanced energy efficiency by enabling a single neuron to employ both fast and modulatory signaling pathways, thereby obviating the need for distinct neuronal groups for each function. Furthermore, it conferred context sensitivity; specifically, peptides facilitate state-dependent regulation without necessitating structural alterations. Finally, it facilitated scale plasticity by enabling gene-regulated peptide expression, which enables long-term adaptation to environmental challenges. Finally, dual signaling systems enabled functional diversity while preserving molecular specialization (Toker et al., 2025). The evolutionary trajectory of neural systems appears to reflect an incremental growth in complexity (Jékely, 2021). Peptide-based volume signaling created a modulatory substrate (Zoli et al., 1999; Fuxe and Borroto-Escuela, 2016), whereas fast synaptic transmission provided temporal precision (Krächan et al., 2017). Cotransmission combined the two, enabling layered communication that supports complex behaviors.

As a result, cotransmission should be regarded as an evolutionary stratification of signaling systems, rather than as a violation of a simple neurotransmission principle. Peptidergic communication predated traditional synaptic transmission and serves as a modulatory framework in modern circuits. The development of SCV-based rapid transmission increased speed and spatial precision but did not remove ancestral peptide systems. Rather, chemical multiplexing across both temporal and spatial dimensions was made possible by evolution’s integration of these dimensions into a single neuron. Because coexistence reflects retained biological logic rather than chance chemical overlap, this evolutionary view helps explain its prevalence.

## 5 Coexistence as a widespread feature of mammalian circuits

**FIGURE 4:**
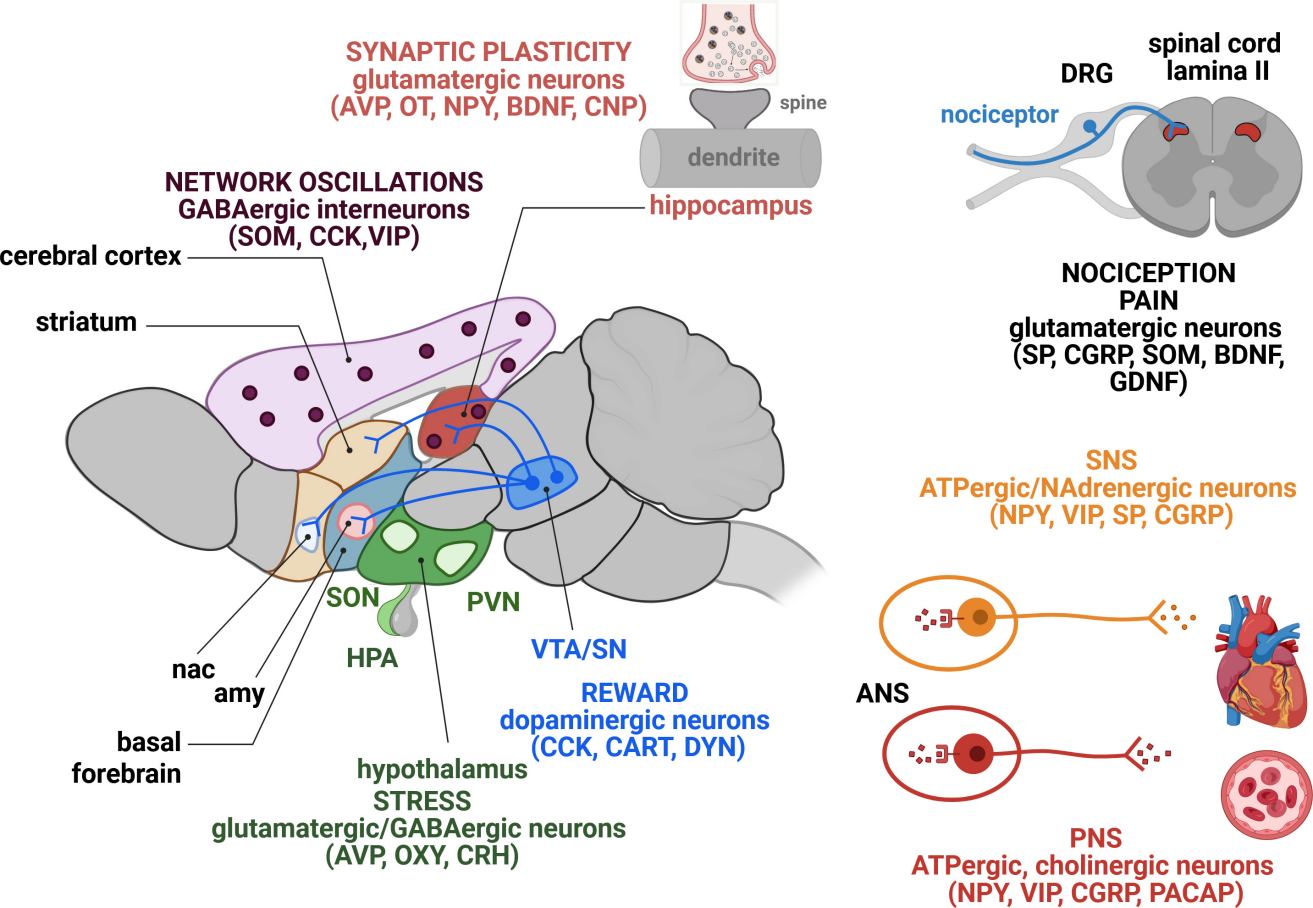
Examples of the functional integration of cotransmission across major neural systems. Representative circuits illustrate how the coexistence of neuropeptides and classical neurotransmitters regulates diverse physiological processes. In cortical and hippocampal networks, peptide-expressing interneurons modulate oscillatory activity and synaptic plasticity. In hypothalamic circuits, cotransmission links neuronal activity to endocrine output under normal and stressful conditions. In rewards pathways, peptides modulate dopaminergic signaling and motivational states. In sensory systems, cotransmission regulates gain control and contributes to central sensitization. In autonomic circuits, the combined release of ATP, monoamines, ACh, and peptides enables graded control of visceral functions (as exemplified by the regulation of the heart and blood vessels). Disruption of these integrated signaling modes contributes to several pathological conditions, including, e.g., chronic pain, stress disorders, addiction, epilepsy, and neurodegeneration. amy, amygdala; ANS, autonomic nervous system; AVP, vasopressin; BDNF, brain-derived neurotrophic factor; CART, cocaine and amphetamine-regulated transcript; CCK, cholecystokinin; CGRP, calcitonin gene-related peptide; CNP, C-type natriuretic peptide; CRH, corticotropin-releasing hormone; DYN, dynorphin; GDNF, glial-derived neurotrophic factor; HPA, hypothalamic–pituitary–adrenal; nac, nucleus accumbens; NPY, neuropeptide Y; OT, oxytocin; PACAP, pituitary adenylate cyclase-activating polypeptide; PNS, parasympathetic nervous system; PVN, paraventricular nucleus; SNS, sympathetic nervous system; SOM, somatostatin; SON, supraoptic nucleus; SP, substance P; VIP, vasoactive intestinal peptide; VTA/SN, ventral tegmental area/substantia nigra. Created in BioRender (2026) https://BioRender.com/k1ppi90.

The evolutionary and molecular logic of cotransmission becomes fully apparent at the level of systems neuroscience. In mammalian brains, neuropeptide–classical transmitter coexistence is not sporadic but pervasive, embedded within circuits that regulate, among others, cognition, emotion, autonomic balance, sensory processing, and endocrine integration (Figure 4). In this section, some of the best-characterized examples of coexistence and cotransmission will be briefly reviewed.

Cortical and hippocampal inhibitory interneurons (Mao and Staiger, 2024) exemplify coexistence within circuit logic. Various GABAergic interneuron subclasses coexpress specific neuropeptides that are associated with their morphology, synaptic targets, and overall network function (Freund and Katona, 2007). For instance, SOM-positive interneurons modulate distal dendritic integration (Takács et al., 2024), whereas CCK- or VIP-containing interneurons affect disinhibitory circuits (Malhotra et al., 2025). Within these cells, GABA mediates rapid inhibitory postsynaptic potentials, while co-released peptides modulate presynaptic transmitter release probability, postsynaptic ionotropic glutamate and GABA receptor phosphorylation, and intrinsic membrane conductance. Consequently, this results in layered inhibition, integrating millisecond-scale synaptic silencing with a more gradual modulation of oscillatory coherence. Furthermore, peptidergic signaling specifically contributes to the tuning of theta and gamma rhythms (Spaak et al., 2012), thereby influencing cognitive functions such as attention and working memory (Hodzic and Riedemann, 2025). Therefore, cotransmission in interneurons extends inhibitory control beyond individual synaptic events, promoting broader network synchronization.

The hippocampus employs distinct signaling mechanisms among neurons to initiate and maintain long-term potentiation (LTP) and long-term depression (LTD) (Bin Ibrahim et al., 2022). Glutamatergic principal neurons may coexpress neuropeptides such as DYN and CCK, whereas hippocampal interneuronal networks contribute peptides including NPY, SOM, VIP, and SP, all of which modulate Ca^2+^ channel activity, N-methyl-D-aspartate (NMDA) receptor function, and kinase cascades (Katona et al., 2017). In addition, peptide signaling affects the shape of dendritic spines, controls dendritic excitability, alters LTP-induction thresholds, and synchronizes astrocytic responses (Patterson and Yasuda, 2011; Guo et al., 2016; Asrican et al., 2020; Honoré et al., 2021). Peptides link transient activity to continuous signaling, thereby promoting the transition from temporary synaptic changes to more permanent changes in cellular structure. Therefore, cotransmission serves as a biochemical link between electrical activity and gene expression.

The mesolimbic pathways and basal ganglia are prime examples of the synergistic and integrative advantages of cotransmission. Dopaminergic neurons in the midbrain frequently coexpress neuropeptides (gastrin-releasing peptide - GRP, NPY, orexins, cocaine- and amphetamine-regulated transcript - CART, and melanin-concentrating hormone - MCH) that modulate stress responses, rewards prediction, and reinforcement learning (Bello et al., 2011). In the striatum, the simultaneous release of peptides affects the activity of medium spiny neurons, the effects of DA receptor signaling, the intracellular dynamics of cAMP, and synaptic plasticity (Yang et al., 2023). Consequently, this integration affects susceptibility to addiction, motivational disorders, and the development of maladaptive habits. Peptides amplify or gate dopaminergic effects depending on firing pattern and physiological context (Buxton et al., 2017), exemplifying the integrated operational model described earlier.

The hypothalamus provides a striking example of functional asymmetry in cotransmission. Many hypothalamic neurons coexpress classical transmitters (glutamate or GABA) with neuropeptides that act both centrally and peripherally (Ludwig and Leng, 2006). Dendritic peptide release can occur independently of standard axonal transmission, generating spatially dissociated signaling areas in a single neuron (Ludwig and Leng, 2006). Magnocellular neurons, which release OT or AVP, can influence local circuit activity and also activate systemic endocrine pathways. Glutamatergic neurons that respond to stress produce CRH (Tasker et al., 2020). Normally, fast synaptic transmission is the main signaling process; however, during stress, sustained firing triggers peptide release. Peptide signaling intervention changes network excitability and activates the hypothalamic–pituitary–adrenal (HPA) axis. This recruitment, which depends on the state of the organism, shows how cotransmission integrates the neural and endocrine systems, allowing circuit activity to drive changes at the organism level.

Primary sensory pathways provide another clear example of how chemical signals depend on the frequency of neuronal firing. In dorsal root ganglion (DRG) neurons that connect to spinal lamina II, glutamate is released in response to weak stimuli (Uenoyama et al., 2021). However, with ongoing or painful stimulation, SP, CGRP, and BDNF are released together with the transmitter amino acid (Ruscheweyh and Sandkühler, 2002). Electron microscopy showed that nociceptive afferents store peptides and neurotrophic factors in LDCVs (Merighi et al., 1991; Bardoni et al., 2007; Salio et al., 2014), which explains how activity alters signal processing. In lamina II, the release of peptides overactivates postsynaptic NMDA receptors, activates microglia and astrocytes, promotes central sensitization, and contributes to chronic pain (Carniglia et al., 2017; Ji et al., 2018). Therefore, cotransmission changes temporary pain signals into lasting hyperexcitability when stimulation continues. In other sensory systems, such as the retina (Brecha et al., 1984; Casini and Brecha, 1991, 1992) and the olfactory bulb (Maher and Westbrook, 2008; Lyons-Warren et al., 2023), peptidergic modulation regulates gain control and temporal filtering, thereby influencing signal-to-noise ratios without altering anatomical connections.

Autonomic neurons are a clear example of hierarchical cotransmission. Sympathetic postganglionic neurons release ATP upon initial activation, NA during moderate activity, and NPY during prolonged, high-frequency stimulation (Cannon et al., 1986; Burnstock et al., 2010). Under various physiological conditions, these three types of messengers regulate, among other things, heart rate and vasoconstriction. Their incremental activation can precisely modulate rapid vasoconstriction, prolonged sympathetic activity, and lasting alterations following stress. In hypertension, e.g., changes in peptide expression can shift the autonomic system toward chronic overactivation (Michel and Rascher, 1995). Similarly, parasympathetic pathways exhibit peptide coexistence, particularly with ACh, involving VIP, PACAP, SP, galanin, and related neuropeptides, which together regulate glandular secretion and smooth muscle function (Michel and Rascher, 1995). Therefore, in both divisions of the ANS, cotransmission allows these circuits to function over different time frames while maintaining a relatively simple structure.

Peptides also affect glial cell function, in addition to their interactions with neurons. Astrocytes and microglia contain neuropeptide receptors, enabling activity-dependent communication between neurons and glia (Carniglia et al., 2017). Peptidergic modulation can control inflammatory responses (Carniglia et al., 2017), synaptic pruning (Nelson et al., 2025), and metabolic support (Nguyen and Dodd, 2024).

## 6 The changing nature of cotransmitter identity: influences from development, activity, and disease

Cotransmission is not a static characteristic. The concurrent presence of neuropeptides and classical neurotransmitters is dynamically regulated throughout development, in response to physiological changes, and during disease (Hökfelt et al., 2018). Unlike classical transmitters, whose synthetic enzymes are often constitutively expressed, neuropeptide production, as mentioned, is tightly coupled to transcriptional programs. This link renders the peptidergic component of cotransmission particularly sensitive to activity, stress, injury, and environmental signals.

### 6.1 Peptide expression developmental dynamics

Neuropeptides are often momentarily expressed during embryonic and early postnatal development (Hevesi et al., 2025). These findings support the assessment that cotransmission contributes to the development of neuronal circuits (Vaaga et al., 2014). Many neurons have broader peptidergic profiles than their adult counterparts during crucial developmental periods. These temporarily synthesized peptides may regulate neuronal migration, stimulate synaptogenesis, promote neurite outgrowth, and influence target selection. They are downregulated or restricted to specific neuronal subpopulations as circuits develop (Harkany et al., 2025). This developmental process suggests that peptides first act as guiding signals during network formation, and then assume roles in controlling activity in fully formed circuits. This is clearly exemplified by the role of BDNF (Barde, 2025), which is not only a well-recognized neurotrophic factor during development (Binder and Scharfman, 2004; Ateaque et al., 2023) but also an important modulator of hippocampal LTP (Mizuno et al., 2003; Lu et al., 2014) and nociceptive synapses (Merighi et al., 2004, 2008; Bardoni and Merighi, 2009). In line with these observations, peptide synthesis in sensory systems is often higher in early development than in adulthood, with synaptic maturation reflecting the system’s greater adaptability during network formation.

### 6.2 Activity-dependent transcriptional reprogramming and remodeling caused by disease and injury

The expression of neuropeptides is greatly affected by neuronal activity. Sustained depolarization, synaptic plasticity, stress exposure, or physical injury can all activate transcription factors such as CREB, activator protein-1 (AP-1, composed of c-Fos/c-Jun), and nuclear factor of kappa light-chain enhancer of B cells (NF-κB) (Wang et al., 2018). All of the above conditions can affect the expression of peptide precursor genes, thereby modifying the peptidergic phenotype of a given neuron. Existing peptide genes can be increasingly transcribed, and previously silent peptide genes may be activated. On the contrary, a reduction in baseline peptide expression may occur, along with coordinated changes in receptor expression. For example, stress can reduce glutamatergic transmission and increase CRH expression in the hypothalamus (Tasker et al., 2020). Another example concerns nociceptive pathways, in which central sensitization is facilitated by sustained stimulation, which increases the production of BDNF and SP in primary afferents (Xu et al., 1992; Kerr et al., 1999; Groth and Aanonsen, 2002). These modifications reshape the modulatory overlay rather than necessarily changing the classical transmitter identity. As a result, the neuron modifies its slow signaling output while maintaining its fast synaptic phenotype.

Significant changes in peptidergic expression are frequently due to peripheral nerve damage, neuroinflammation, and neurodegenerative diseases. Axotomy or inflammation can change the number and distribution of LDCVs, promote *de novo* peptide expression in non-peptidergic neurons, and enhance the production of SP (Weissner et al., 2006), CGRP (Palkovits, 1995), or BDNF (Tonra et al., 1998) in DRG neurons. Normally, primary afferent neurons release glutamate to signal acute harmful stimuli. Persistent or intense stimulation triggers LDCV exocytosis, leading to the co-release of SP, CGRP, and BDNF in the dorsal horn lamina II (Uenoyama et al., 2021, 2024). Synapses formed by primary afferents in the dorsal horn change following injury, as shown by ultrastructural studies, and this remodeling links structural modifications to functional hypersensitivity (Matthews et al., 1992). There are several other examples of remodeling of peptide expression in neurons, e.g., chronic stress modifies peptidergic signaling in limbic networks (Maita et al., 2022), and addiction-related plasticity entails recalibration of dopaminergic tone via peptide-mediated mechanisms (Lüscher and Malenka, 2011). Also, network hyperexcitability in epilepsy may be exacerbated or compensated for by changes in neuropeptide production in inhibitory interneurons (Kovac and Walker, 2013). Therefore, depending on context and time span, cotransmitter plasticity can be either detrimental or adaptive.

### 6.3 Subcellular compartmentalization and local translation

Some neurons may control peptide availability by translating mRNA locally in dendrites or axons (Jung et al., 2012; Ludwig et al., 2016; Herbison, 2021). Direct demonstrations of neuropeptide local translation are rather uncommon compared with classical somatic synthesis, but several important studies support local dendritic/axonal translation of neuropeptide-related mRNAs. AVP is one of the clearest examples. Its mRNA was shown to localize to the dendrites of hypothalamic magnocellular neurons and undergo local translation (Mohr and Richter, 2004). Related work on OT/AVP magnocellular neurons has demonstrated dendritic trafficking of neuropeptide precursor mRNAs and the local translational machinery associated with secretory activity (Biever et al., 2019). Subcellular regulation of precursor mRNA stability or translation could fine-tune release capacity, even though most neuropeptides are produced in the soma and transported along processes within LDCVs (Sossin and Scheller, 1991). The possible compartment-specific release of different neurotransmitters further complicates cotransmission. Dendritic peptide release occurs in certain neurons independently of classical axonal release (Ludwig and Pittman, 2003). Because dendrites mediate modulatory feedback within local networks, whereas axon terminals are primarily responsible for rapid synaptic output, compartmentalization of release leads to signaling asymmetry (Ludwig et al., 2016). Remarkably, neurons can control incoming and outgoing information streams differently, specifically because of this spatial compartmentalization.

### 6.4 Homeostatic and metaplastic control

Cotransmission also contributes to homeostatic plasticity (Pozo and Goda, 2010). Peptide expression can modify circuit gain, restoring stability when network activity persists. For instance, sustained downregulation of inhibitory peptides may improve responsiveness, whereas overexpression may promote hyperexcitability (Demarque and Spitzer, 2012). Metaplasticity, the plasticity of synaptic plasticity, augments neural communication, extending its effects beyond simple synaptic scaling (Peineau et al., 2018). This phenomenon can modulate gene expression, thereby impacting synaptic receptor types and excitability thresholds.

Furthermore, alterations in peptide transcription may influence the system’s response to conventional neurotransmitters (Pratelli and Spitzer, 2025). Consequently, cotransmitter plasticity modifies both ongoing signaling and the fundamental principles that dictate future plasticity. Therefore, cotransmitter plasticity demonstrates that neuromessenger coexistence is a dynamic characteristic of gene-regulatory networks. Neurons can alter peptide expression during development, reprogram peptidergic output in response to activity, alter vesicular composition following damage, activate compartment-specific release, and support both maladaptive and homeostatic adaptations. Classical neurotransmitters primarily mediate synaptic functions, whereas neuropeptides exert longer-lasting modulatory influences that shape synaptic plasticity and network adaptation. Thus, cotransmission links short-term activity to long-term phenotypic change by functioning at the nexus of electrophysiology and transcriptional regulation.

## 7 Pathophysiological consequences of cotransmission

### 7.1 The detrimental effects of chemical multiplexing

The above-discussed properties that make cotransmission an efficient adaptation process–temporal stratification, gene-regulated plasticity, and network-wide modulation–also leave it susceptible to dysregulation (Figure 4). In various neurological and psychiatric disorders, pathological alterations of peptidergic neurons modify circuit excitability, plasticity thresholds, and homeostatic equilibrium. Cotransmission thus operates at a critical interface between adaptive regulation and disease propagation, as illustrated by the following examples.

Nociceptive pathways provide a clear example of how cotransmission can have negative effects on pain circuitry. The shift from the self-protective short-lasting glutamatergic signaling to sustained peptidergic modulation indicates a transition from defensive nociception to pathological central sensitization (Ji et al., 2018). Several outcomes follow peptide recruitment, including enhanced NMDA receptor function, increased intracellular Ca^2+^ accumulation, and the activation of microglia and astrocytes (Latremoliere and Woolf, 2009). As a consequence, activation of pro-inflammatory mediators (Mashaghi et al., 2016; Pinho-Ribeiro et al., 2017; Zhu and Bhatia, 2023) and LTP (Willis, 2002; Ruscheweyh et al., 2011) follows at nociceptive synapses. Injury-induced alterations in LCDV density and peptide expression, which reinforce hyperexcitability even after peripheral damage has subsided, further enhance neuronal and circuit function (Walters et al., 2023). Remarkably, a distinct population of DRG neurons that express SOM and GDNF acts antagonistically, thereby reducing nociceptive input to higher centers (Salio et al., 2014; Merighi, 2016, 2017, 2021; Ferrini et al., 2021).

As mentioned previously, dopaminergic neurons within mesolimbic circuits, which are crucial for rewards perception, motivation, and reinforcement learning (Bello et al., 2011), co-express several neuropeptides that regulate synaptic plasticity and DA-modulated behavioral strategies (Dunigan and Roseberry, 2022). Importantly, the glucagon-like peptide-1 receptor 1 (GLP-1R) mediates this modulation, receiving GLP-1 projections from the nucleus of the solitary tract and the intermediate reticular nucleus in the caudal hindbrain (Merkel et al., 2025). Following repeated drug exposure, sustained burst firing enhances peptide release, consequently altering downstream receptor signaling and intracellular cascades. Peptide modulation can increase dopamine receptor sensitivity and alter intracellular signaling pathways, including cAMP-dependent protein kinase (PKA) and extracellular signal-regulated kinase (ERK) cascades, alter synaptic strength in striatal medium spiny neurons, and affect how rewards prediction errors are processed (Iigaya et al., 2019; Shao et al., 2021). These changes then contribute to compulsive drug-seeking behavior and a decrease in behavioral flexibility. As a result, cotransmission is implicated not only in acute reinforcement but also in the long-term reprogramming of motivational circuits. Furthermore, the continued presence of peptidergic signaling could stabilize harmful synaptic states, which would make addiction resistant to extinction.

Peptide cotransmission is a common phenomenon in stress-responsive hypothalamic and limbic circuits. In anxiety and depression, the dysregulation of peptide release disturbs the balance between excitatory and inhibitory neurons (Tao et al., 2024). Such an imbalance results in network hyperresponsiveness or maladaptive stress responses. Several peptides, including CRH, NPY, GRP, and OT, may be dysregulated under different pathophysiological conditions (Rana et al., 2022). Extended stimulation of peptidergic transmission can effectively “lock” neural circuits into a changed equilibrium, thereby diminishing their resilience and adaptive flexibility. Excess CRH over-activates the HPA axis, acting as a powerful modulator that shifts the balance toward fear and anxiety.

Conversely, numerous instances demonstrate that diminished peptidergic signaling can also yield adverse consequences. While neuropeptide NPY is typically viewed as protective, reduced activity or dysfunction during chronic stress can diminish its inhibitory effects, thereby impairing its capacity to mediate stress resilience (Enman et al., 2015). Furthermore, a deficiency in GRP release in the prefrontal cortex (PFC) correlates with elevated anxiety, as this peptide typically regulates fear-related responses (Goto et al., 2022). In addition, decreased OT levels can fail to inhibit CRH neurons, leading to increased anxiety and elevated cortisol secretion under stress (Kuppusamy et al., 2021).

Interneurons in the cortex and hippocampus, which typically express inhibitory peptides and low-molecular-weight neurotransmitters, are crucial for maintaining neural network function (Bin Ibrahim et al., 2022). SOM, e.g., can reduce excitability at both pre- and postsynaptic levels by altering the probability of neurotransmitter release from the presynaptic neuron and the postsynaptic neuron’s response (Katona et al., 2017). Modifications in interneuron peptide expression have been documented, encompassing both compensatory overexpression and pathogenic downregulation, depending on the stage and brain region affected in epilepsy (Marx et al., 2013). Disruption of peptidergic modulation can lower seizure threshold, enhance synchronous firing, and destabilize oscillatory coherence. Because cotransmission affects oscillatory dynamics, its impairment could convert temporary excitatory bursts into continuous epileptiform activity (Drexel and Sperk, 2022).

Chronic stress or hypertension is associated with increased expression and altered release of NPY from sympathetic neurons, which, as mentioned, also contain NA and ATP. Changes in NPY levels lead to prolonged vasoconstriction and sustained sympathetic neuron activation (Robertson et al., 2023). As a result, the ANS shifts from a state of plastic adjustment to a state of constant overactivity. The slow kinetics of peptide signaling may contribute to the chronicity of autonomic dysfunction, making reversal more difficult than modulation of the NA fast transmitter system alone.

Peptidergic signaling also influences neuron–glia interactions. In neurodegenerative diseases, altered peptide release can influence microglial activation, astrocytic metabolic support, synaptic pruning, and the availability of neurotrophic factors (Zhang et al., 2026). A decrease in the support that peptides such as NPY and CRH, acting on synaptic maintenance and astrocyte-dependent pruning, provide could make neurons more vulnerable (Futran-Sheinberg et al., 2025). In contrast, excessive signaling from inflammatory peptides, e.g., SP, might hasten degeneration (Carniglia et al., 2017).

### 7.2 A brief note on cotransmission as a therapeutic target

The properties of cotransmission discussed in the previous sections offer some therapeutic opportunities (McGonigle, 2012; Todaro et al., 2023). Targeting peptide receptors offers several advantages, including greater specificity due to restricted receptor expression, slower receptor kinetics that may reduce excitotoxic risk, and the possibility to modulate rather than completely suppress fast synaptic transmission (Tang et al., 2026). For these reasons, peptide receptor antagonists or agonists are being evaluated for chronic pain (Rahman et al., 2026), migraine (de Prado and Russo, 2006), stress disorders (Chaki and Kanuma, 2012), and metabolic diseases (Bailey et al., 2023). In contrast, the pleiotropic and state-dependent nature of peptidergic signaling requires precise targeting to avoid disrupting adaptive modulation. Future therapies could be envisaged from a better understanding of the operational models of cotransmission–hierarchical, parallel, conditional, or integrated–to design interventions that rebalance rather than silence neural circuits.

In summary, disease states frequently involve a shift in the balance between fast synaptic transmission and slower peptidergic modulation. Because peptide expression is transcriptionally regulated and activity-dependent, maladaptive changes can persist long after the initiating stimulus. Cotransmission thus acts as both amplifier and stabilizer of circuit states. When appropriately regulated, it enhances flexibility and resilience. However, its dysregulation leads to several diseases (Leite et al., 2017). These issues are disturbances of chemical multiplexing rather than mere synaptic irregularities, a clear concept when cotransmission is properly considered a dynamic, stratified signaling system.

## 8 Methodological advances enabling cotransmission research

The recognition of cotransmission as a fundamental organizational principle of the nervous system was closely associated with methodological and technical advancements. Progress in ultrastructural analysis, molecular profiling, live imaging, and circuit manipulation has deepened our understanding of the complexity of cotransmission. Each technological advance has moved the conceptual framework from mere anatomical features to functional integration and dynamic plasticity.

The initial demonstration of transmitter coexistence and costorage relied on immunohistochemistry and immunoelectron microscopy. Dual and triple immunogold labeling methods enabled visualization of neuropeptides and classical neurotransmitters within the same neuronal profile (Merighi et al., 1988; Merighi and Polak, 1993; Merighi, 2002, 2009). Ultrastructural analyses yielded conclusive evidence of segregation into SCVs and LDCVs within individual boutons. These observations established the morphological foundation for differential release. High-resolution investigations elucidated costorage patterns in primary sensory afferents and other systems, demonstrating, in addition, the concurrent presence of peptides and neurotrophic factors within LDCVs (Salio et al., 2014; Merighi, 2016) and associating vesicular plasticity with pathological conditions. While crucial, ultrastructural immunolabeling is limited by its reliance on images of fixed tissue and by the potential for epitope masking. Nonetheless, it remains crucial for demonstrating vesicular segregation and spatial correlations with nanoscale precision.

Single-cell RNA sequencing (scRNA-seq) has fundamentally changed our understanding of cotransmission. This method allows one to study individual cells within a population, rather than averaging signals across whole groups of neurons, providing unprecedented resolution into cotransmission patterns. Transcriptomic studies have shown that many neurons express genes for both the usual neurotransmitter systems and several neuropeptide precursors (Smith et al., 2019). These transcriptomic datasets also reveal that peptide expression is highly specific to each cell type, that distinct interneuron subclasses are defined by peptide signatures, that injury or stress induces transcriptional reprogramming of peptidergic genes, and that previously unrecognized peptide coexpression patterns exist. Importantly, transcriptomics distinguishes between potential and genuine cotransmission. mRNA presence does not guarantee vesicular packaging or release, but it defines the molecular capacity for coexistence. Combining scRNA-seq with spatial transcriptomics now allows mapping peptide identity and tissue distribution together, bridging molecular profiling with circuit regional anatomy.

Targeted manipulation of genetically defined neuronal populations has enabled the functional examination of cotransmission (Dao et al., 2019). Researchers can isolate the effects of specific cotransmitter combinations by expressing light-sensitive ion channels or engineered receptors within peptide-defined neurons. Furthermore, optogenetic stimulation, when coupled with pharmacological blockade of peptide receptors, permits the experimental dissociation of fast synaptic transmission from slower modulatory influences (Zhang et al., 2019). This approach has clarified certain aspects of frequency-dependent peptide recruitment, some behavioral roles of cotransmission, and circuit-specific modulation. However, short stimulation patterns are frequently used in traditional optogenetic paradigms, which may underestimate peptidergic contributions by failing to trigger LDCV release (Arrigoni and Saper, 2014). Therefore, it is essential to create stimulation protocols that replicate natural burst firing.

Advancements in genetically encoded fluorescence sensors for neuromodulators and neurotransmitters have created new opportunities for *in vivo* monitoring of release dynamics (Patriarchi et al., 2020; Ravotto et al., 2020). Peptide detection poses significant technological challenges due to slower kinetics and lower extracellular concentrations, whereas sensors for traditional neurotransmitters such as glutamate and dopamine are well-established. Total internal reflection fluorescence (TIRF) imaging and super-resolution microscopy (Midorikawa, 2018) have enabled visualization of individual vesicular fusion events and differentiation of SCV and LDCV exocytosis based on size and fusion characteristics (Kreutzberger et al., 2019). These methods show distinct release thresholds and kinetics in real time. Very recently, a genetically encoded OT sensor based on G-protein-coupled receptor activation (GRAB), GRABOT1.0, was described. GRABOT1.0 enables imaging of OT release *ex vivo* and *in vivo* with suitable sensitivity, specificity, and spatiotemporal resolution (Qian et al., 2023). Two other genetically encoded tools for investigating peptidergic transmission in behaving mice were also developed: a LDCV sensor (CybSEP2) that detects presynaptic neuropeptide release and a silencer (NEP_LDCV_) that specifically degrades neuropeptides within LDCVs (Kim et al., 2024). The future development and exploitation of peptide-specific optical sensors will be crucial for quantifying the spatial diffusion and temporal persistence of peptidergic signaling in intact circuits.

Mass spectrometry-based proteomics has emerged as a powerful tool for characterizing the molecular makeup of vesicle populations (Al-Hasani et al., 2018; Girven et al., 2022). By isolating LDCVs and subsequently performing proteomic analyses, researchers can gain insights into the intricate composition of their contents, including the various auxiliary proteins that play roles in their maturation and transport. Emerging single-vesicle proteomics and nanoscale flow cytometry approaches may determine whether molecularly distinct LDCV subtypes exist within a single neuron. Such a resolution would directly address unresolved questions about the specialization of selective peptide packaging and release (see also Section “9 Unresolved questions and future directions: toward a unified theory of chemical multiplexing”).

Mathematical models integrating Ca^2+^ dynamics, vesicle pools, receptor kinetics, and diffusion parameters have been used to simulate cotransmission over time (Loewenstein and Sompolinsky, 2003; Bielecki et al., 2008; Gandolfi et al., 2021). These models predict the circumstances in which peptides dominate network behavior and show how firing-frequency thresholds translate into non-linear chemical output. Although it remains challenging, incorporating peptidergic modulation into large-scale network simulations is essential for understanding disease states, oscillatory control, and plasticity induction. Tools that can resolve peptide release with the same temporal precision as that currently available for classical transmitters will be necessary for future advancements. These approaches will help clarify whether cotransmission relies on dynamically changing operational modes or rigid hierarchies.

## 9 Unresolved questions and future directions: toward a unified theory of chemical multiplexing

Cotransmission is still poorly understood at the mechanistic, systems, and computational levels despite decades of research. Although coexistence has been documented and operational models have been identified, basic problems such as vesicle specialization, release selectivity, circuit integration, and therapeutic exploitation remain to be addressed. Addressing these issues will determine whether cotransmission can be fully integrated into mainstream computational and translational neuroscience.

### 9.1 Are dense-core vesicles functionally heterogeneous?

One of the central unresolved questions concerns the molecular identity of LDCVs within individual neurons. While costorage of multiple peptides within a single vesicle is well documented, it remains unclear whether distinct LDCV subpopulations contain different peptide combinations, or whether vesicle identity encodes functional specialization, or whether release probability varies among vesicle subclasses. In *Lymnea stagnalis*, cell-type-specific sorting of neuropeptides has been identified as a mechanism to modulate the peptide composition of LDCVs (Klumperman et al., 1996). However, similar observations have been more difficult to replicate in vertebrates. Single-vesicle proteomics and super-resolution imaging may determine whether LDCVs are homogeneous storage units or molecularly stratified compartments. Functional heterogeneity would imply a higher degree of chemical selectivity than currently appreciated.

### 9.2 Is selective peptide release possible?

Conventional models assume that all peptides within a vesicle are released simultaneously. However, partial fusion (“kiss-and-run”) or regulated pore dilation could, in theory, permit differential discharge of cargo (Rutter and Tsuboi, 2004). It remains to be critically assessed whether specific peptides can be preferentially released under defined Ca^2+^ conditions, whether vesicle fusion mode influences peptide composition of the extracellular signal, and whether certain peptides are retained within partially fused vesicles. Experimental tools capable of directly visualizing peptide-specific release kinetics are needed to resolve these issues.

### 9.3 How is cotransmission integrated into neural coding frameworks?

Current models of neural computation emphasize spike timing, firing rate, and synaptic weights. Cotransmission introduces an additional dimension: chemical composition of output. Future theoretical frameworks must incorporate frequency-dependent chemical transitions, spatial diffusion fields, GPCR signaling kinetics, and transcriptional feedback loops. Incorporating these variables into computational models may reveal that cotransmission expands the coding dimensionality beyond classical rate- or temporal-coding schemes (Makadia et al., 2015).

### 9.4 How does cotransmission interact with glial networks?

Peptide signaling frequently transcends synaptic boundaries, influencing astrocytes and microglia. Nevertheless, the extent to which glial responses reciprocally modulate neuronal cotransmission remains unclear. The potential for glial cells to modulate peptide degradation or diffusion, the impact of glial activation on LDCV release thresholds, and the influence of chronic neuroinflammation on cotransmitter identity remain unresolved (Mazon et al., 2026). Understanding neuron–glial integration will be essential for linking cotransmission to neurodegenerative and inflammatory disorders.

### 9.5 What determines the operational mode in cotransmission?

As discussed previously, neurons may operate under hierarchical, parallel, conditional, or integrated cotransmission models depending on functional conditions (Svensson et al., 2019). The molecular determinants of these operational states remain unknown. Ca^2+^ buffering capability, vesicle docking protein composition, receptor density and distribution, transcriptional status, and metabolic environment are examples of potential regulators. Identifying these characteristics would elucidate the mechanisms underlying shifts in circuits between modulatory dominance and rapid, precise signaling.

### 9.6 Therapeutic targeting: modulation without disruption

Peptide receptors, with their restricted expression and regulatory functions, present appealing therapeutic targets (Bali et al., 2014). However, the pleiotropy and context-dependence inherent in peptidergic signaling present challenges for therapeutic interventions. Future therapeutic approaches should prioritize targeting receptor subtypes within specific circuit nodes, utilizing biased agonism to fine-tune intracellular signaling pathways, and combining molecular targeting with activity-dependent interventions to re-establish balanced cotransmission, rather than simply blocking individual transmitters. Precision neuromodulation may necessitate conceptualizing cotransmission not as additive signaling, but as integrated chemical logic.

### 9.7 Toward a unified conceptual framework

Cumulative evidence suggests that cotransmission is not an exception to classical neurotransmission but its expansion. A unified framework should consider dual vesicular architecture as a structural substrate, differential Ca^2+^ sensitivity as an activity integrator, temporal stratification as functional layering, transcriptional plasticity as an adaptive amplifier, and systems integration as a network stabilizer. Such a framework positions cotransmission as a scalable regulatory system capable of converting transient activity into sustained circuit states.

## 10 Conclusion: cotransmission as a core computational principle of neural systems

The coexistence of neuropeptides and classical neurotransmitters represents far more than a historical correction to an oversimplified interpretation of Dale’s principle. It reflects a deeply conserved organizational strategy that endows neurons with multilayered chemical output. Through dual vesicular architecture, differential Ca^2+^ sensitivity, spatial divergence of release sites, and transcriptionally regulated plasticity, cotransmission enables temporal and functional stratification of signaling across milliseconds to hours.

From an evolutionary perspective, peptide-based modulation preceded rapid synaptic transmission. Rather than being displaced by the emergence of fast amino acid–mediated signaling, this ancestral modulatory layer was integrated into the same cellular framework. Modern neurons therefore operate as chemical multiplexers, combining precise point-to-point transmission with diffuse, state-dependent modulation. This layered architecture enhances computational dimensionality without increasing anatomical complexity.

Oscillatory coherence, plasticity thresholds, motivational states, endocrine integration, sensory gain control, and autonomic balance are all regulated at the systems level by cotransmission. It links chemical identity to spike frequency, enabling assessment of both signal composition and strength from firing patterns. The transfer of electrical code into molecular diversity increases the coding capacity of neural circuits. Importantly, peptidergic expression plasticity links short-term activity to long-term phenotypic alteration. Cotransmission connects structural remodeling and electrophysiological processes via activating transcriptional pathways. Transcriptional activation is crucial for maintaining balance and adaptability in a healthy state.

In contrast, when peptide signaling goes wrong, it can lead to harmful circuit states in various diseases, resulting in neurodegeneration, chronic pain, addiction, stress disorders, epilepsy, and autonomic dysfunction. Recent technological advances, such as single-cell transcriptomics, circuit manipulation, and ultrastructural studies, have clarified the extent and complexity of cotransmission. However, important questions remain about how best to target therapies, how to incorporate cotransmission into computational models, the differences between vesicles, and the specific mechanisms of neurotransmitter release. Multiscale strategies that connect molecular events to network dynamics and behavior will be necessary to close these gaps.

This review’s main conclusion is that cotransmission should no longer be considered an anomaly or a biological curiosity. It is a basic principle of nervous system computation. Neurons acquire context sensitivity, adaptive resilience, and scalable control by incorporating slow modulatory signaling into fast synaptic architecture. Chemical multiplexing must therefore be included as a fundamental aspect of circuit operation in any future theoretical, experimental, or translational frameworks of nervous function. Determining the stability and fragility of complex nervous systems will require understanding how neurons dynamically balance the levels of classical neurotransmitters and peptides.

## Footnotes

1
https://www.nobelprize.org/prizes/medicine/1977/summary/
